# Tuning Transition Metal 3d Spin state on Single‐atom Catalysts for Selective Electrochemical CO_2_ Reduction

**DOI:** 10.1002/adma.202417034

**Published:** 2025-03-13

**Authors:** Yipeng Zang, Yan Liu, Ruihu Lu, Qin Yang, Bingqing Wang, Mingsheng Zhang, Yu Mao, Ziyun Wang, Yanwei Lum

**Affiliations:** ^1^ Department of Chemical and Biomolecular Engineering National University of Singapore Singapore 117585 Republic of Singapore; ^2^ School of Chemical Sciences University of Auckland Auckland 1010 New Zealand; ^3^ Institute of Materials Research and Engineering (IMRE) Agency for Science Technology and Research (A*STAR) 2 Fusionopolis Way, Innovis #08‐03 Singapore 138634 Republic of Singapore

**Keywords:** CO2 reduction, electrocatalysis, single‐atom, spin state

## Abstract

Tuning transition metal spin states potentially offers a powerful means to control electrocatalyst activity. However, implementing such a strategy in electrochemical CO_2_ reduction (CO_2_R) is challenging since rational design rules have yet to be elucidated. Here we show how the addition of P dopants to a ferromagnetic element (Fe, Co, and Ni) single‐atom catalyst (SAC) can shift its spin state. For instance, with Fe SAC, P dopants enable a switch from low spin state (*d*_x2‐ y2_^0^, *d*_z2_^0^, *d*_xz_^2^, *d*_yz_^1^, *d*_xy_^2^) in Fe‐N_4_ to high spin state (*d*_x2‐y2_^0^, *d*_xz_^1^, *d*_yz_^1^, *d*_z2_^1^, *d*_xy_^2^) in Fe‐N_3_‐P. This is studied using a suite of characterization efforts, including X‐ray absorption spectroscopy (XAS), electron spin resonance (ESR) spectroscopy, and superconducting quantum interference device (SQUID) measurements. When used for CO_2_R, the SAC with Fe‐N_3_‐P active sites yields > 90% Faradaic efficiency to CO over a wide potential window of ≈530 mV and a maximum CO partial current density of ≈600 mA cm^−2^. Density functional theory calculations reveal that high spin state Fe^3+^ exhibits enhanced electron back donation via the *d*_xz_/*d*_yz_‐π* bond, which enhances ^*^COOH adsorption and promotes CO formation. Taken together, the results show how the SAC spin state can be intentionally tuned to boost CO_2_R performance.

## Graphical abstract

The introduction of P into a single‐atom Fe catalyst is shown to induce a shift in its electronic configuration toward a high spin state. This is proposed to enhance electron back donation via the *d*_xz_/*d*_yz_‐π* bond, which improves *COOH adsorption and facilitates the electrochemical conversion of CO_2_ to CO.

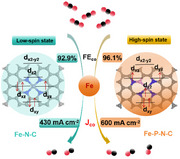

## 1 Introduction

Rising global energy demands have led to massive fossil fuel consumption and serious concerns over climate change issues. There is, therefore, an urgent need to develop technologies that can effectively store, capture, and utilize carbon emissions. For this purpose, renewable electricity‐powered electrochemical CO_2_ reduction (CO_2_R) has attracted interest as a potential pathway toward realizing an artificial carbon cycle.^[^ ^1^, ^2^ ^]^ Interestingly, depending on the type of catalyst employed for CO_2_R, a wide variety of value‐added products such as CO, formate, and ethylene can be generated.^[^ ^3^ ^]^

To this end, there has been significant interest in the development of single‐atom catalysts (SACs) for facilitating CO_2_ conversion to CO.^[^ ^4^ ^]^ Such SACs typically consist of single metal atoms dispersed onto a conductive substrate such as nitrogen‐doped carbon. Often, these single metal atoms are anchored onto the substrate by coordinating together with pyridinic nitrogen to form active sites with configurations such as M‐N_4_. For instance, Jiang et al. screened a range of different transition metal SACs (Ni, Fe, Co, Mn, and Cu) and found Ni SAC to exhibit the highest Faradaic efficiency (FE) to CO.^[^ ^5^ ^]^ Later works by Zhang et al. and Fan et al. then investigated the impact of Ni SAC active site coordination (Ni‐N_3_‐C, Ni‐N_2_‐C) on the CO_2_R performance.^[^ ^6^, ^7^ ^]^ Recently, our group also found that tuning the nanocurvature of the carbon substrate can be used to control the CO_2_R performance of SACs.^[^ ^8^ ^]^

Although significant progress has been achieved in these works, several key issues remain. While a high FE to CO of >90% has been achieved using SACs in numerous reports, the operating voltage window for optimal performance is often rather narrow. Another issue is that the maximum CO partial current density is limited to <500 mA cm^−2^ because the hydrogen evolution reaction (HER) tends to dominate at large applied overpotentials. These issues thus motivated us to pursue and explore new design strategies that could be complementary for further improving SAC performance.

Here, we hypothesized that tuning the spin state of transition metal SACs could offer a new degree of freedom for rational design and optimization.^[^ ^9^ ^]^ Notably, such a concept has been mainly explored in the realm of oxygen evolution/reduction,^[^ ^10^, ^11^, ^12^, ^13^ ^]^ while its applications for CO_2_R have thus far been limited to the design of molecular catalyst systems.^[^ ^14^, ^15^ ^]^ For instance, Ding et al. used thermal annealing to switch Co phthalocyanine from a low to a high spin state, resulting in an increase in the electron backdonation of CO via the *d*_xz_/*d*_yz_−2π* bond. This weakened the C─O bond strength, which facilitated an improved selectivity for methanol production.^[^ ^14^ ^]^ Work by Wang et al. found that hydrazine treatment could shift Ni phthalocyanine to a higher spin state with more unpaired 3*d* electrons, resulting in improved ^*^COOH adsorption.^[^ ^16^ ^]^ This then enhanced the conversion of CO_2_ to ^*^COOH and increased CO selectivity.

**Figure 1:**
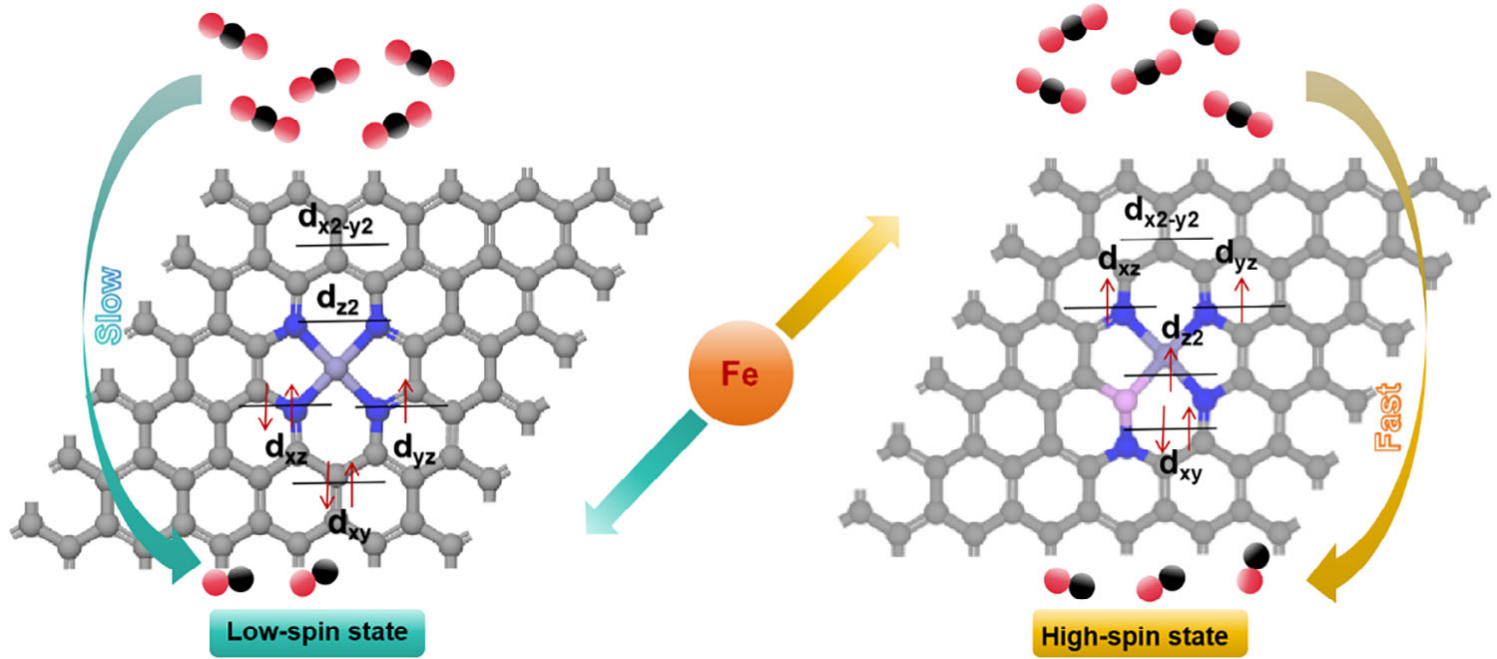
The illustration of catalyst configuration. Left is Fe─N─C with low‐spin state Fe(III) center and right is Fe─P─N─C with high‐spin state Fe(III) center. Atomic colors are as follows. Blue: N, red: O, purple: P, black/gray: C, violet: Fe.

Motivated by these reports, we sought to develop a suitable strategy to regulate the spin state of transition metal SACs. In this work, we discovered that the addition of P dopants to Fe SACs enables a shift from a low spin state in Fe─N─C to a high spin state in Fe─P─N─C, which significantly improves the CO_2_R activity and selectivity toward CO production (**Figure** **1**). A suite of characterization efforts, including Fe L‐edge X‐ray absorption spectroscopy (XAS), electron spin resonance spectroscopy (ESR), and superconducting quantum interference device (SQUID) measurements, provide evidence for the electronic configuration change from a low spin to a high spin state.

CO_2_R measurements show that the high spin state Fe─P─N─C catalyst exhibits enhanced CO selectivity as compared to Fe─N─C. Density functional theory (DFT) calculations reveal that the high spin state in Fe─P─N─C has an increased number of unpaired electrons, which promotes the adsorption for *COOH due to enhanced electron back donation via the *d*_xz_/*d*_yz_‐π* bond. We further find that this strategy can also be applied to Co and Ni SACs, with P dopants similarly enabling a shift toward high spin state configurations that display enhanced CO_2_R selectivity and activity toward CO production.

## 2 Results and Discussion

**Figure 2:**
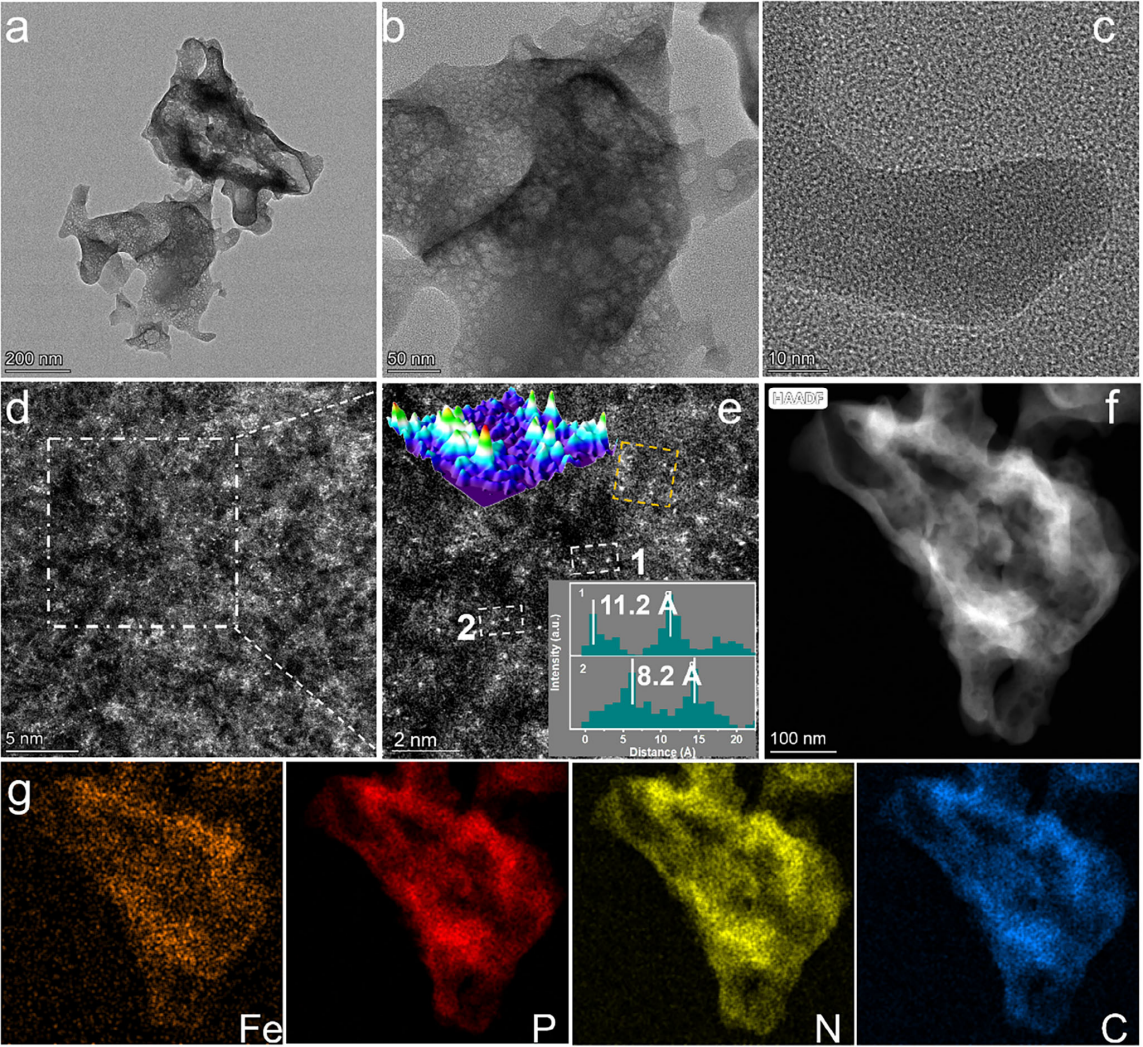
Morphology characterization of Fe─P─N─C. a,b) TEM and c) HRTEM images. d) AC‐ADF‐STEM image. e) The selected amplification area in (d), the insets are a 3D atomic energy intensity diagram (top left corner) along the yellow dotted line and the intensity profiles (bottom right corner) of areas 1 and 2. f,g) HAADF‐STEM image and corresponding EDS element maps of Fe, P, N, and C.

We synthesized the Fe─N─C and Fe─P─N─C SAC samples by mixing the metal nitrate precursor with melamine and melamine phosphate, respectively, followed by thermal annealing at 900 °C under an inert gas environment (see Experimental Section for more details). X‐ray diffraction (XRD) measurements in Figure S1 (Supporting Information) show the absence of metallic Fe signals and only one broad peak at ≈22° that is associated with the carbon support. Transmission electron microscopy (TEM) and high‐resolution transmission electron microscopy (HRTEM) images of Fe─P─N─C (**Figure** **2**a–c) display no obvious particles and lattice fringes. Aberration‐corrected high‐angle annular dark‐field scanning transmission electron microscopy (AC‐HAADF‐STEM) in Figure 2d demonstrates the uniform and singly dispersed nature of the Fe single atoms. Similar observations were made for the Fe─N─C catalyst (Figure S2, Supporting Information).

The isolated nature of the Fe atoms of Fe─P─N─C was further studied as shown in a 3D atomic energy intensity diagram along the yellow dotted line and the intensity profiles of X‐Y (inset in Figure 2e). The measured distances between two Fe atoms in Fe─P─N─C exceed their atomic size, which verifies its single‐atom nature. Energy‐dispersive X‐ray spectroscopy (EDS) mapping shows that the elements Fe, N(P), and C are uniformly distributed throughout both the Fe─N─C and Fe─P─N─C catalysts (Figure 2f,g; Figure S2d, Supporting Information). The Fe loading of each catalyst was determined using inductively coupled plasma mass spectrometry (ICP‐MS) and found to be ≈1.67 and 1.80 wt.% for Fe─N─P─C and Fe─N─C, respectively (Table S1, Supporting Information).

Raman spectroscopy was also performed, where distinct results were obtained for Fe─N─C and Fe─P─N─C (Figure S3, Supporting Information). The spectra indicate that Fe─P─N─C has a slightly higher degree of defects than Fe‐N─C as observed in the increased ratio of I_D_/I_G_. This is likely a result of the larger radius of P relative to that of C and N. In addition, Fe─P─N─C displays new Raman modes: A_1__g_ caused by the anti‐symmetric bending vibration of P‐N‐C and B_1__g_ due to the symmetric bending vibration of C─N─C.^[^ ^17^, ^18^ ^]^ XPS measurements also show the presence of Fe, N(P), and C in the samples (Figures S4 and S5, Supporting Information). The N 1*s* narrow scan of Fe─N─C was deconvoluted into five peaks: pyridinic N, metal N bonding, pyrrolic N, graphitic N, and oxidized N.^[^ ^19^, ^20^, ^21^ ^]^ In comparison, only pyridinic N and metal N were present in Fe─P─N─C. Similar results were obtained for both Fe SACs in the Fe 2*p* narrow scan, where signals related to that of Fe^2+^, Fe^3+,^ and satellite peaks could be observed.^[^ ^19^, ^20^ ^]^ For Fe─P─N─C, the P *2p* narrow scan confirms the existence of P─C and P─N bonds in the sample.^[^ ^20^, ^21^ ^]^

**Figure 3:**
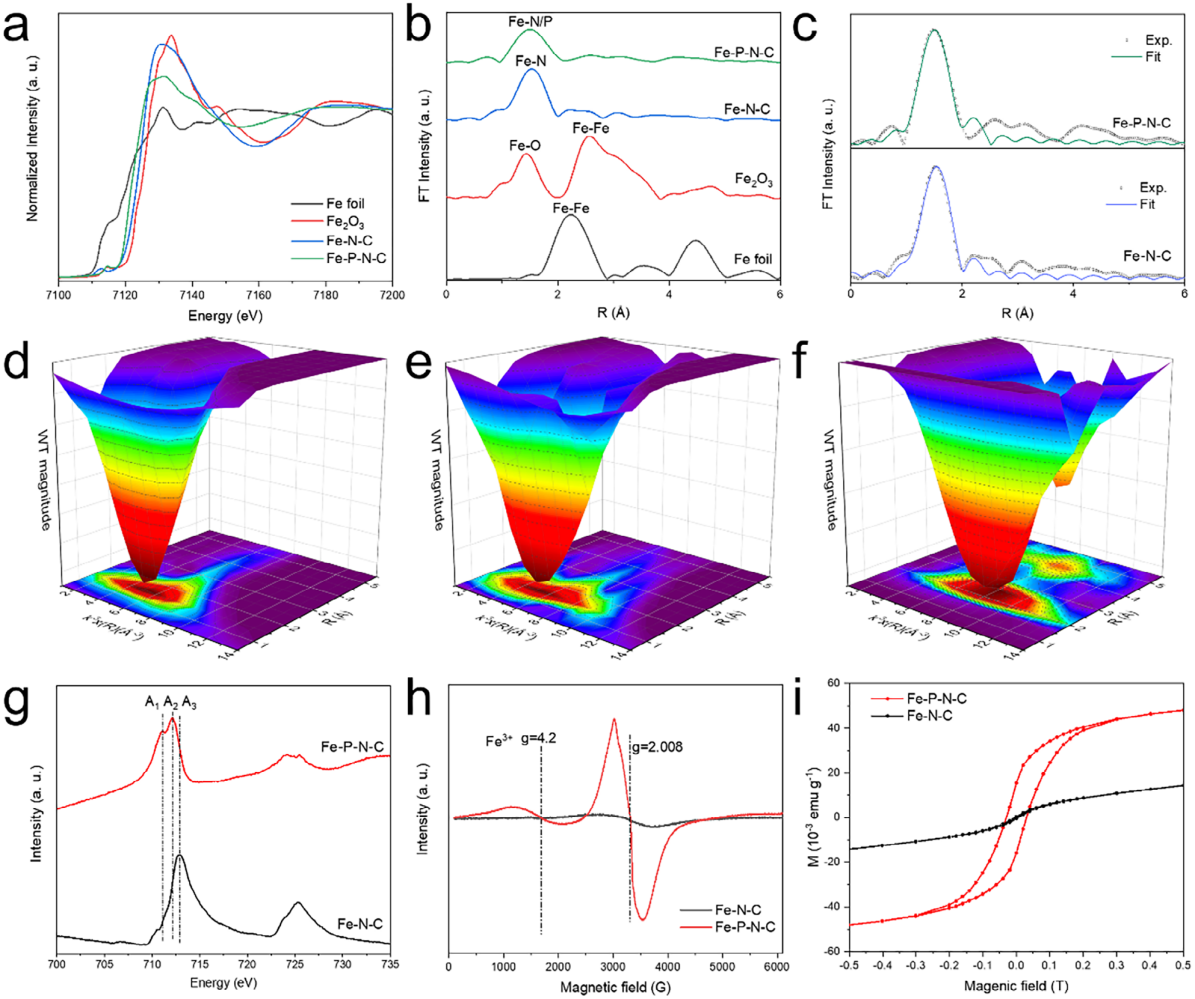
Coordination and spin electron structure characterization. a) Fe K‐edge XANES and b) the corresponding FT R‐space spectra of Fe foil, Fe─N─C, Fe─P─N─C, and Fe_2_O_3_. c) R‐space fitting curves of Fe─N─C and Fe─P─N─C. d–f) The wavelet transform spectra of Fe foil (d), Fe─N─C (e) and Fe─P─N─C (f). g) Fe L‐edge XAS spectra of Fe─N─C and Fe─P─N─C. h) Electron Spin Resonance spectra and i) magnetic field dependence of magnetization (M‐H) curves of Fe─N─C and Fe─P─N─C.

X‐ray absorption near‐edge structure (XANES) and extended X‐ray absorption fine structure (EXAFS) were further used to study the electronic and coordination structure of both catalysts. As shown in **Figure** **3**a, the near‐edge adsorption for Fe SACs is located between that of Fe foil and Fe_2_O_3_. This indicates that the oxidation states of Fe in both samples are between Fe^0^ and Fe^3+^,^[^ ^22^ ^]^ consistent with the XPS results. Fourier transform extended X‐ray absorption fine structure spectroscopy spectra (EXAFS) in Figure 3b show a dominant peak located at ≈1.5 Å for Fe─N─C, which can be assigned to Fe─N coordination.^[^ ^23^ ^]^ Importantly, no Fe─Fe peak is observed in both Fe─N─C and Fe─P─N─C as compared to Fe foil, indicating that the Fe species in both catalysts are dispersed as isolated atoms.^[^ ^24^, ^25^ ^]^ We note that Fe─P─N─C exhibits similar features to Fe─N─C but a wider scatter path at ≈1.5 Å, which is likely due to contribution from Fe─P bonding.^[^ ^25^ ^]^

Wavelet transform (WT) analysis further confirms the atomic dispersion of Fe species in both Fe─N─C and Fe─P─N─C, in which no Fe‐Fe vectors are observed with only an intensity maximum attributed to Fe─N and Fe─P in the first shell (Figure 3d–f; Figure S6, Supporting Information).^[^ ^25^ ^]^ The results of EXAFS fitting (Figure 3c; Table S2, Supporting Information) indicate that isolated Fe atoms are each coordinated by four N atoms to form a Fe‐N_4_ configuration for Fe─N─C, while a different Fe‐N_3_‐P configuration exists in Fe─P─N─C. Hence, this result suggests that the electronic structure of the Fe single atoms in Fe─P─N─C is different from Fe─N─C, potentially also shifting the spin state.

To investigate this further, we conducted Fe L_2,3_ edge XAS measurements (Figure 3g), which are more indicative of spin states. The spectra can be divided into the L_2_ and L_3_ regions, which are associated with the transition from 2*p*_1/2_ and 2*p*_3/2_ levels to unoccupied 3*d* orbitals respectively. Three features appear in the energy region of the Fe L_3_‐edge, denoted as A_1_, A_2,_ and A_3_. The A_1_ peak can be assigned to the transition from 2*p*_3/2_ to the 3*d*_z2_ orbital, while the peaks A_2_ and A_3_ originate from the transition to 3*d*_x2‐y2_ orbitals.^[^ ^26^ ^]^ With Fe─P─N─C, we find that the L_2,3_ edge becomes shifted to lower binding energies, with a significant increase in the A_1_ peak as compared to Fe─N─C. This suggests that the electronic configuration of Fe^3+^ in Fe─P─N─C is quite different from Fe^3+^ in Fe─N─C. According to prior literature,^[^ ^27^, ^28^, ^29^ ^]^ Fe^3+^ in Fe─N─C will typically exist in the ground state with ^2^A_1__g_ symmetry (*d*_x2‐y2_^0^, *d*_z2_^0^, *d*_xz_,_yz_^3^, *d*_xy_^2^), which is low spin (S = 1/2). However, with distortion of the coordination environment, the ground state electron configuration can switch to (*d*_x2‐y2_^0^, *d*_xz,yz_^2^, *d*_z2_^1^, *d*_xy_^2^), which is high spin (S = 3/2). In our case, we reasoned that this distortion is induced by the Fe─P bonding present in Fe─P─N─C.

ESR spectroscopy was also performed on the catalysts, which is a technique that is sensitive to the presence of unpaired electrons. As shown in Figure 3h, a typical characteristic signal of high‐spin Fe^3+^ with rhombic zero field splitting is detected at g = 4.2 (S = 3/2), which appears to be stronger on Fe─P─N─C as compared Fe─N─C. In addition, a broad axial signal at g = 2.008 indicates a quasi‐octahedral or square pyramidal coordination structure.^[^ ^30^ ^]^ The ESR spectroscopy results suggest that P incorporation increases the number of unpaired electrons, which changes the spin state of single atom Fe from low spin to high spin, consistent with L_2,3_‐edge analysis.

We also further examined the magnetic effects induced by the electronic spin state through superconducting quantum interference device (SQUID) measurements. Based on the hysteresis loop (M‐H) obtained (Figure 3i), both Fe─N─C and Fe─P─N─C exhibit ferromagnetism.^[^ ^11^, ^12^ ^]^ Importantly, the saturation magnetization (Ms) and coercivity (u) of Fe─P─N─C are higher than Fe─N─C, which indicates the presence of more unpaired Fe 3d electrons that give rise to a larger magnetic moment.^[^ ^12^, ^31^ ^]^ Hence, the above results reveal that P coordination can indeed change the spin state of the Fe single atom.

**Figure 4:**
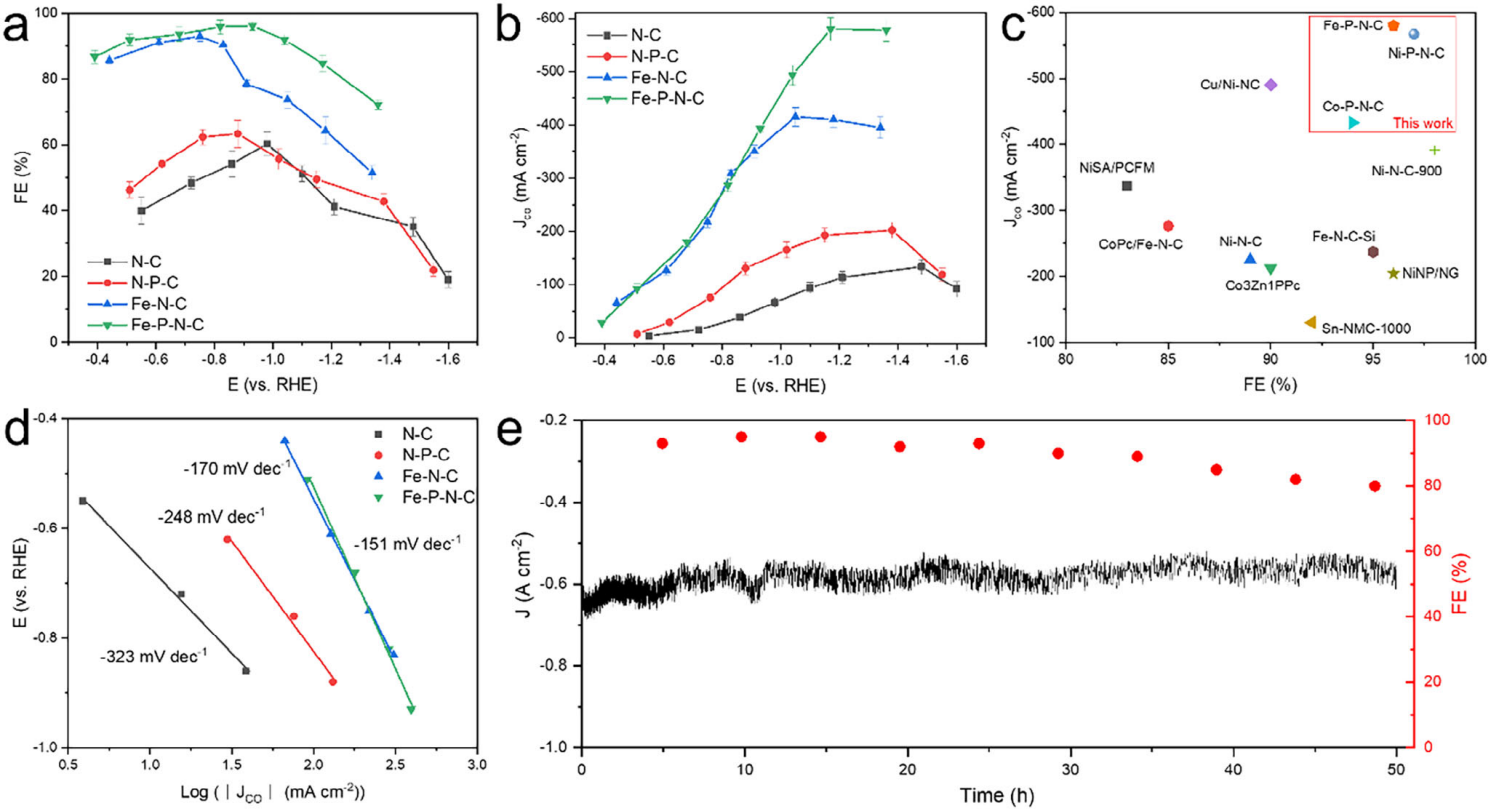
Electrochemical CO_2_R performance. a) FE_CO_ and b) J_CO_ of N─C, P─N─C, Fe─N─C and Fe─P─N─C. c) The comparison of SAC samples tested in this study with the literature reported SACs. d) Tafel slopes derived from J_CO_ of N─C, P─N─C, Fe─N─C, and Fe─P─N─C. e) Stability test of Fe─P─N─C.

Having established the successful switch toward a higher spin state with P incorporation, we then proceeded to evaluate and compare the CO_2_R performance of Fe─N─C and Fe─P─N─C. These experiments were performed in a flow cell system using a gas diffusion electrode (GDE) with continuous CO_2_ flow through a gas chamber (Figure S7a, Supporting Information). The reference electrode was Ag/AgCl, and an IrO_x_‐coated Ti mesh was used as the counter electrode. Control samples (without any Fe loading) consisting of thermally annealed melamine (N─C) and melamine phosphate (P─N─C) were also synthesized, and their CO_2_R performance was tested for comparison. The obtained FEs toward CO as a function of potential for Fe─P─N─C and Fe─N─C are shown in **Figures** **4**a and S7b–e (Supporting Information).

First, we found that both Fe─N─C and Fe─P─N─C exhibit higher CO activity and selectivity than the N─C and P─N─C control samples, indicating that single Fe atoms indeed serve as active sites. The maximum FE to CO (FE_CO_) of 96.1% is obtained for Fe─P─N─C, which is higher than that of Fe─N─C (92.9%). Notably, the potential window where FE_CO_ is >90% is −0.51 to −1.04 V versus RHE, much wider than that of Fe─N─C which is from −0.61 to −0.83 V versus RHE. As for the CO partial current density (J_CO_) in Figure 4b, Fe─P─N─C exhibits a higher J_CO_ than Fe─N─C across the whole potential window, with a maximum value of ≈600 mA cm^−2^. Notably, this value is higher than most reported SACs in the literature (Figure 4c; Table S3, Supporting Information).

We then measured the double‐layer capacitance (C_dl_) using cyclic voltammetry in order to estimate and compare electrochemically active surface areas (ECSA). As shown in Figure S8a–c (Supporting Information), the C_dl_ of Fe─P─N─C is 25.4 mF cm^−2^, which is comparable to that of Fe─N─C with a value of 20.6 mF cm^−2^. Hence, this indicates that the improved CO_2_R performance of Fe─P─N─C is not due to surface area effects. Electrochemical impedance spectroscopy (EIS) at −1.0 V versus RHE also indicates that Fe─P─N─C exhibits lower charge transfer resistance as compared to Fe─N─C (Figure S8d, Supporting Information).^[^ ^7^ ^]^

The J_CO_ derived Tafel curves were also plotted as shown in Figure 4d. The Tafel slope of ≈151 mV dec^−1^ for Fe─P─N─C is lower than Fe─N─C with a value of ≈170 mV dec^−1^. This indicates that Fe─P─N─C allows for enhanced reaction kinetics toward CO production as compared to Fe─N─C.^[^ ^6^, ^7^ ^]^ We also tested Fe─P─N─C over an extended 50 h duration with an applied potential of −1.17 V versus RHE (Figure 4e). We obtained a FE_CO_ of ≈95% in the initial 15 h and a slight decay to ≈92% in the following 20 h. A gradual decay to ≈80% was observed near the end period, which we attribute to the gradual flooding of the gas diffusion layer rather than catalyst deactivation. Post‐experiment characterization of the catalyst was then carried out with TEM, AC‐HAADF‐STEM, Fe K‐edge XAS, Fe L‐edge XAS, and ESR spectroscopy (Figures S9–S16, Supporting Information). From these results, we observed no significant changes in the structure, morphology, and Fe─P─N─C active sites after long‐term CO_2_R electrolysis, indicating reasonable catalyst stability.

The Fe─P─N─C catalyst was also integrated into a 4 cm^2^ membrane electrode assembly (MEA) system with an anion‐exchange membrane. An Ir‐coated Ti mesh anode was employed, along with 0.1 m KOH as the anolyte (Figure S17, Supporting Information). In this system, a high FE_CO_ of 96.6% was obtained at −500 mA cm^−2^ with Fe─P─N─C and a value of FE_CO_ >90% over a current density range of −100 to −600 mA cm^−2^.

We also sought to understand if the P doping strategy could be generalized and applied to other transition metal SACs. We thus synthesized a series of other first‐row transition metal SACs consisting of Mn, Co, Ni, and Zn. For each transition metal element (M), both the M─N─C and M─P─N─C catalysts were synthesized and extensively characterized (Figures S18–S85, Supporting Information). Notably, we found that the Co and Ni SACs exhibited similar results to Fe SAC, where P incorporation induced switching to a higher spin state resulting in a stronger magnetic moment. In contrast, for Mn and Zn SACs, we found that P incorporation did not result in a change in spin state and an increase in the magnetic moment. This is likely due to the electron spin cancellation effect of Mn *d* orbitals and fully filled Zn *d* orbitals.^[^ ^32^, ^33^ ^]^ When we performed CO_2_R tests, we obtained higher FE_CO_ and J_CO_ values with P incorporation for the Co and Ni SACs. On the other hand, P incorporation did not significantly influence the CO_2_R performance of Mn and Zn SACs. Importantly, these results are consistent with our working hypothesis that a higher spin state has beneficial effects on the CO_2_R performance of SACs.

**Figure 5:**
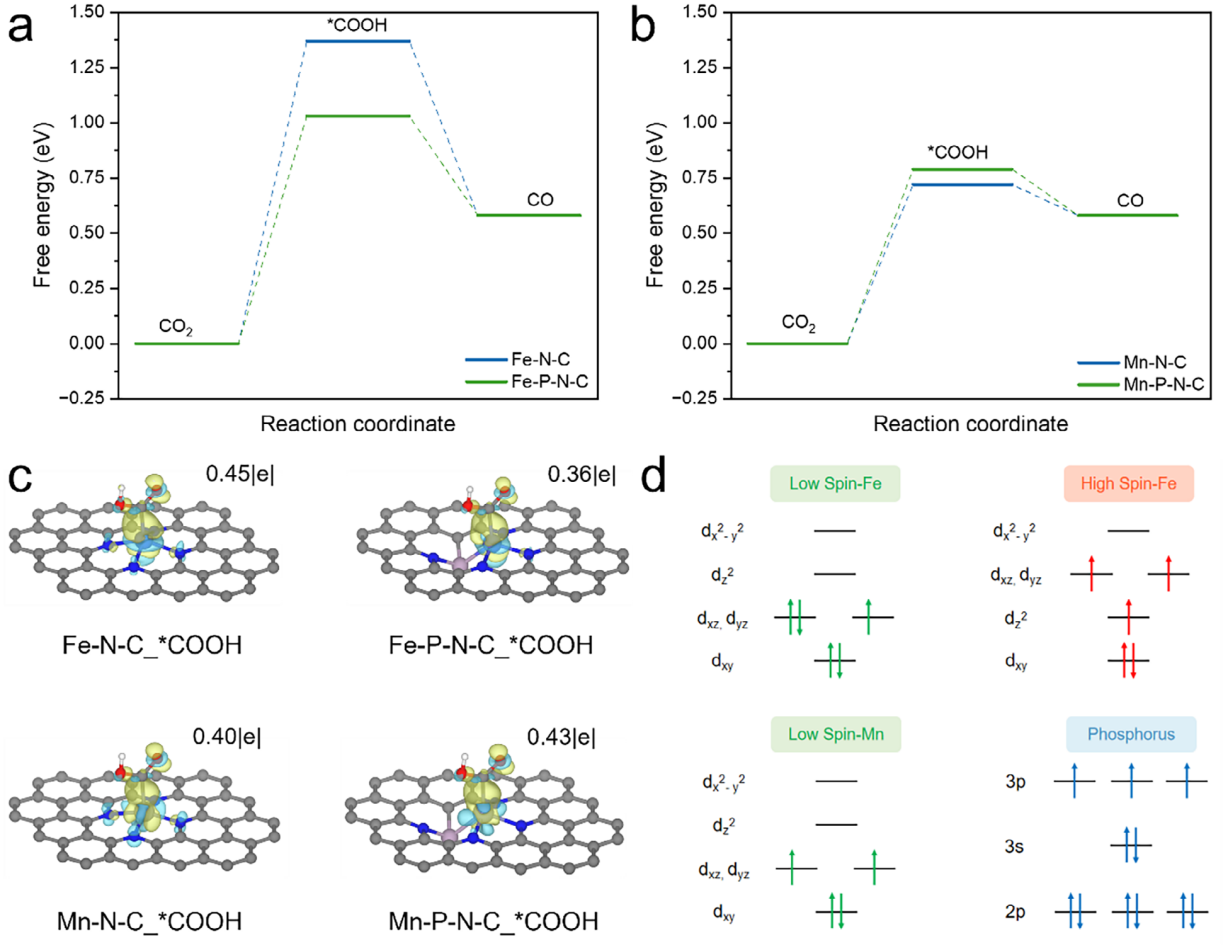
DFT calculations. a) Free energy diagrams of CO_2_R on Fe─N─C and Fe─P─N─C. b) Free energy diagrams of CO_2_R on Mn‐N─C and Mn─P─N─C. c) Charge density difference (CDD) of ^*^COOH on Fe─N─C, Fe─P─N─C, Mn─N─C and Mn─P─N─C. The isosurface level was set to 0.004 e/bohr^3^ (yellow: electron accumulation; cyan: electron depletion). The violet, purple, pink, blue, gray, and white balls represent Fe, Mn, O, N, C, and H atoms respectively. d) Orbital diagrams for low spin and high spin states of Fe and Mn, and electronic configuration of P atom.

Finally, we performed DFT calculations to shed light on how P incorporation improves the CO_2_R performance of Fe SAC. As a comparison, similar calculations were performed on Mn SAC, which experimentally did not exhibit increased CO_2_R activity with P incorporation. All DFT models are shown in Figures S86–S100 (Supporting Information). The DFT calculations results reveal that P incorporation significantly enhances the performance of Fe SACs, while having a weak effect on Mn SACs. Specifically, P atom doping decreases the ΔG of the potential‐determining step (PDS): CO_2_ → ^*^COOH from 1.37 to 1.03 eV for Fe SAC, which improves the CO_2_R activity (**Figure** **5**a; Tables S5 and S6, Supporting Information). This performance enhancement is associated with the change in spin state of the Fe single atom induced by P atom doping, which appears to enhance ^*^COOH adsorption. From in situ attenuated total reflectance surface‐enhanced infrared absorption spectroscopy (ATR‐SEIRAS) experiments, we also found that Fe─P─N─C exhibits enhanced adsorption of ^*^COOH (Figure S102, Supporting Information), which is consistent with the DFT calculations (Figure 5a).

In comparison, the ΔG change in the PDS for CO_2_R on Mn SAC is minimal, which increases from 0.72 to 0.79 eV with P incorporation. This suggests that P incorporation has a limited impact on the CO_2_R performance of Mn SAC (Figure 5b; Tables S7 and S8, Supporting Information). In summary, the DFT results are consistent with the experimental results which indicate that P doping has a weak influence on the CO_2_R performance of Mn SACs as compared to the beneficial impact on Fe SACs.

Further calculations of electron transfer show significant changes in the ^*^COOH electron transfer characteristics due to P atom doping. For Fe SAC, the incorporation of the P atom reduces the electron transfer from 0.45 to 0.36 e for ^*^COOH, which is likely due to a decrease in the energy level gap, facilitating electron backdonation via the d_xz_/d_yz_‐π* bond. As for Mn SACs, the electron transfer to ^*^COOH increases slightly from 0.40 to 0.43 e, indicating that the impact of the P atom on electron transfer is minor and correlates with the limited improvement in CO_2_R performance (Figure 5c; Figure S103, Supporting Information). This is consistent with P doping having a larger impact on the electronic configuration of the Fe single atom.

Furthermore, the analysis of the d‐band center further elucidated the effect of P doping on the catalytic performance of Fe and Mn SACs. For Fe SACs, the d band center shifts significantly from −0.708 eV (Fe─N─C) to −0.933 eV (Fe─P─N─C) (Figure S104, Supporting Information). P doping affects the d‐band center of Fe and optimizes the catalytic performance by changing its local coordination environment and electronic distribution. In particular, the electronegativity of P atoms triggers the redistribution of surrounding charges, forming a new electric field effect around Fe and promoting the electronic coupling of ^*^COOH. P doping also triggers the spin state of Fe to change from low spin to high spin, and this spin state change enhances the reactivity of Fe sites. In contrast, for Mn SACs, the d‐band center shifts from −1.062 eV (Mn─N─C) to −1.003 eV (Mn─P─N─C), indicating that P doping has a relatively small effect on the Mn electronic structure. (Figure S105, Supporting Information) This limited shift is related to the small changes observed in the free energy change and electron transfer of the Mn SACs. These d‐band center trends further support the conclusion that P doping exerts a stronger effect on the electronic structure of Fe single atoms compared to Mn.

The electronic orbital configuration diagrams illustrate changes in the Fe SACs from low to high spin states (Figure 5d). In the low spin state, the electronic configuration 3*d* (d_x2‐ y2_^0^, d_z2_^0^, d_xz_^2^, d_yz_^1^, d_xy_^2^) exhibits a low magnetic moment. After P incorporation, the Fe 3*d* electronic configuration changes to Fe 3*d* (d_x2‐y2_^0^, d_xz_^1^, d_yz_^1^, d_z2_^1^, d_xy_^2^), which is a high spin state with a larger magnetic moment. We postulate that this change in spin state is primarily due to the weakening of the crystal field and the reduction in energy level splitting caused by P atom doping, which shifts the electronic configuration of Fe from paired low spin to unpaired high spin. As for Mn, there are two unpaired electrons in the 3*d* orbitals in the ground state. The P dopant does not significantly alter the spin state of Mn, resulting in the less sensitive response of Mn to changes in the crystal field compared to Fe. This is likely due to the high energy gap between d_xy_ and d_z2_, which makes it less susceptible to external changes in the crystal field.

## 3 Conclusion

In summary, L_2,3_ edge XAS, ESR, and M‐H curves analysis shows that Fe─P─N─C has a high spin state as compared to Fe─N─C with a low spin state. This was found to improve the J_CO_ and potential window where >90% FE_CO_ was obtained. Using DFT calculations, we found that P incorporation results in the RDS energy change decreasing by 0.34 eV from 1.37 to 1.03 eV. Interestingly, we found that this P doping strategy could also induce switching to a higher spin state for Co and Ni SACs, with a concomitant improvement in CO_2_R performance. This study provides new insights into the role of spin state regulation in designing SACs with enhanced CO_2_R performance.

## 4 Experimental Section

### Materials

Manganous nitrate (Mn(NO_3_)_2_.4H_2_O), Ferric nitrate (Fe(NO_3_)_2_.9H_2_O), Cobalt nitrate (Co(NO_3_)_2_.6H_2_O), Nickel nitrate (Ni(NO_3_)_2_.6H_2_O) and Zinc nitrate (Zn(NO_3_)_2_.6H_2_O), Iridium oxide (IrO_x_) were purchased from Sigma Aldrich. Melamine, melamine phosphate, potassium hydroxide, and Nafion ionomer solution (5 wt.%) were purchased from Macklin Chemicals (Shanghai, China). The gas diffusion layer (GDL, YLS‐30T, thickness 0.19 mm), Ti mesh (thickness 1 mm), and nickel foam (thickness 1 mm) were purchased from Sinero Tech. Co., Ltd. (Suzhou, China). Anion exchange membrane (NEOSEPTA, AHA) was purchased from Tokuyama Corp. The Ag/AgCl reference electrode was purchased from Gauss Union (Wuhan, China).

### Preparation of M‐N and M‐P Mixture

M‐N mixture was prepared using the following method. Typically, 100 mg of the metal precursor nitrate was dissolved into 20 mL of deionized water and ultrasonicated for 30 min to form solution A. 2.0 g of melamine was then dissolved into 30 mL of deionized water to form solution B. Then, solution A was added to solution B and allowed to sit for another 60 min. The mixture was then stirred for 12 h, followed by overnight freeze drying to obtain a dry white powder. The M‐P mixture was prepared by replacing the melamine precursor with melamine phosphate.

### Preparation of M‐N/C and M‐P‐N‐C

100 mg as‐synthesized M‐N or M‐P mixture was put into a quartz crucible and transferred to a tube furnace. M‐N‐C or M‐P‐N‐C was obtained by annealing at 900 °C for 3.0 h under N_2_ flow with a flow rate of 100 mL min^−1^.

### Physicochemical Characterizations

X‐ray diffraction (XRD) patterns were collected at a Panalytical X'pert PPR diffractometer with a Cu Kα radiation source (λ = 1.5418 Å) at 40 kV and 40 mA. Transmission electron microscopy (TEM), high resolution transmission electron microscopy (HRTEM) were measured at an FEI TALOS F200X with an acceleration voltage of 200 kV. High‐angle annular dark‐field scanning transmission electron microscopy (HAADF‐STEM) images were recorded by using a FEI Themis Z scanning/transmission electron microscope operated at 300 kV, equipped with a probe spherical aberration corrector. Raman measurement was conducted on a Horiba LabRam 352 Odyssey Nano Raman Spectrometer system. The mass loading of metal ions was measured on an Inductively Coupled Plasma mass spectrometry (ICP‐MS, Thermo Scientific iCAP7600). X‐ray photoelectron spectroscopy (XPS) measurements were carried out on Thermo Scientific ESCSLAB 250i X‐ray Photoelectron Spectrometer, using Al Kα radiation source, with C 1 s (284.6 eV) as a calibration reference. Hard X‐ray absorption spectrum (XAS) measurements were operated at the XAFCA beamline of the Singapore Synchrotron Light Source. Soft XAS measurements were conducted at the photoemission end‐station at beamline (BL10B) in the National Synchrotron Radiation Laboratory (NSRL, Hefei, China). Electron spin resonance (ESR) spectra were performed on the steady high magnetic field facilities in the High Magnetic Field Laboratory (HFIPS), Chinese Academy of Sciences (Hefei, China). Magnetic measurements were carried out by using a superconducting quantum interference device (SQUID) in the High Magnetic Field Laboratory (HFIPS), Chinese Academy of Sciences (Hefei, China).

### Preparation of Gas Diffusion Electrodes (Working Electrode)

The ink was first prepared. Typically, ∼10 mg of powder samples were dispersed in a 1.0 mL mixture solution of water, ethanol, and Nafion ionomer (volume ratio of 7:2:1). After the mixture was ultrasonically dispersed for 60 min, ≈800 µL inks were dropped onto the microporous layer of carbon paper with a size of 2.0 cm * 2.0 cm to prepare the working electrode. The mass loading was controlled at ≈2.0 ± 0.1 mg cm^−2^.

### CO_2_R Measurements

CO_2_R measurements were operated at a home‐made flow cell. Commercial Ag/AgCl electrode and IrO_x_ coated Ti mesh were employed as reference and counter electrodes, respectively. The GDE loaded catalyst was used as the working electrode, while 1.0 m KOH was used as the electrolyte solution. Electrolysis was conducted by using an electrochemical workstation (AutoLab PGSTAT204). The flow rates of CO_2_ and electrolyte solution were controlled at 30 and 5.0 mL min^−1^, respectively. All potentials were referenced to a reversible hydrogen electrode (RHE) and corrected by iR compensation according to the formula: E (RHE) = E (Ag/AgCl) + 0.21 V + (0.0591 × pH) – (iR × 85%). The MEA electrolyzer was assembled using a graphite flow field plate for CO_2_ feeding and current collection at the cathode, as well as a titanium flow field plate for aqueous solution feeding and current collection at the anode. A fresh catalyst coated cathode, an Ir black‐coated Ti mesh anode, and an anion exchange membranes Alkymer (QAPPT) were used for each electrolysis test. The in situ attenuated total reflectance‐surface enhanced infrared absorption spectroscopy (ATR‐SEIRAS) measurements were performed on a Nicolet iS50 FTIR spectrometer (Thermo Fisher Scientific).

### Product Analysis

The gas products were analyzed by an on‐line gas chromatography (Agilent, GC 890). The Faradaic efficiency (FE) of H_2_ and CO are calculated as follows: FE = (n×f×c×F)/(I×N),

Where, n: electron transfer number; f: gas flow rate; c: concentration of the detected gas product; F: Faraday constant, 96485 C mol^−1^; I: total current; N: the unit molar volume of gas, 22.4 L mol^−1^.

The partial current density of a specific product (J) is calculated: J = J_total_ * FE.

### Computational Details

All calculations in this work were carried out with the Perdew‐Burke‐Ernzerhof (PBE)^34^ functional using the Vienna Ab initio Simulation Package (VASP).^[^ ^34^, ^35^ ^]^ The project‐augmented wave (PAW) method was used to represent the core‐valence interaction.^[^ ^36^, ^37^ ^]^ For the calculations of total energy, a cut‐off energy of 500 eV was set for plane wave basis sets to expand the valence electronic states. Spin polarization was included in all calculations to obtain an accurate description of electronic structures. The D3BJ correction method^[^ ^38^ ^]^ was employed in order to include van der Waals (vdW) interactions. For optimization, k‐point sampling with a (3 × 3 × 1) mesh within the Monkhorst‐Pack scheme was utilized, as the length of the slab cell is 14.80 × 14.80 × 10.71Å^3^. A vacuum layer of 10 Å was applied to avoid lateral interactions, and the geometry optimization and energy calculations were finished when the electronic self‐consistent iteration and force reached 10^−5^ eV and 0.05 eV Å^−1^, respectively.

The CO_2_R and HER could occur in the following basic steps:

(1) $$CO_{2}\left(g\right)+H^{+}+e^{-}+*\to *\mathrm{COOH}$$

(2) $$*\mathrm{COOH}+H^{+}+e^{-}\to *\mathrm{CO}+H_{2}O\left(l\right)$$

(3) $$H^{+}+e^{-}+*\to H*$$

(4) $$H*\to *+1/2H_{2}$$

where * denotes the active sites on the catalyst surface.

### The Gibbs Free Energy Change

The Gibbs free energy change (ΔG) of each elemental step was calculated based on the computational hydrogen electrode (CHE) method developed by Nørskov et al.^[^ ^39^ ^]^ Under standard conditions (T = 298.15 K, pH = 0, and U = 0 V (vs SHE)), the ΔG is defined by the following equation. Based on this, the free energy of OER is calculated as follows:

(5) $$\Delta G_{\mathrm{ads}}=\Delta E_{\mathrm{ads}}+\Delta E_{\mathrm{ZPE}}-T\Delta S_{\mathrm{ads}}$$

where *∆E_ads_* is the electronic adsorption energy, *∆E_ZPE_* is the zero‐point energy difference between adsorbed and gaseous species, T is the temperature (298.15 K) in the above reaction system, and Δ*S* represents the difference in the entropies between the adsorbed state and gas phase. Charge density and differences are calculated using Bader code.^[^ ^40^ ^]^ For energy correction of the result, use the energy of OH is H_2_O‐1/2H_2_. The source of entropy is the NIST Chemistry Webbook (https://webbook.nist.gov/chemistry/). The electronic binding energy is referenced as 1/2 H_2_ for each H atom, plus the energy of the clean slab. The zero‐point energy of the CO_2_R intermediates can be found in Table S4 (Supporting Information).

## Conflict of Interest

The authors declare no conflict of interest.

## Author Contributions

Y.Z. and Y.L. contributed equally to this work. Y.Lum and Z.W. supervised the project. Y.Z. conceived the idea, designed and carried out the experiments. Y.Liu, Y.M. and R.L. performed the computational work. Z.W. supervised the computational work. Q.Y and B.W. supported the catalyst development. M.Z. carried out the XPS measurements. Y.Z. and Y.Lum co‐wrote the manuscript. All authors discussed the results and assisted during the manuscript preparation.

## Supporting information



Supporting Information

## Data Availability

The data that support the findings of this study are available from the corresponding author upon reasonable request.

## References

[1] G. Wang , J. Chen , Y. Ding , P. Cai , L. Yi , Y. Li , C. Tu , Y. Hou , Z. Wen , M. Li , Chem. Soc. Rev. 2021, 50, 4993. 33625419 10.1039/d0cs00071j

[2] Y. Zang , P. Wei , H. Li , D. Gao , G. Wang , Electrochem. Energy Rev. 2022, 5, 29.

[3] M. Ross , P. Luna , Y. Li , C.‐T. Dinh , D. Kim , P. Yang , E. Sargent , Nat. Catal. 2019, 2, 648.

[4] D. Gao , T. Liu , G. Wang , B. X. , ACS Energy Lett. 2021, 6, 713.

[5] K. Jiang , S. Siahrostami , T. Zheng , Y. Hu , S. Hwang , E. Stavitski , Y. Peng , J. Dynes , M. Gangisetty , D. Su , K. Attenkofer , H. Wang , Energy Environ. Sci. 2018, 11, 893.

[6] Y. Zhang , L. Jiao , W. Yang , C. Xie , H. Jiang , Angew Chem. Int. Ed. 2021, 60, 7607. 10.1002/anie.202016219 33432715

[7] Q. Fan , P. Hou , C. Choi , T.‐S. Wu , S. Hong , F. Li , Y.‐L. Soo , P. Kang , Y. Jung , Z. Sun , Adv. Energy Mater. 2020, 10, 1903068.

[8] B. Wang , M. Wang , Z. Fan , M. a. C. , S. Xi , L.‐Y. Chang , M. Zhang , N. Ling , Z. Mi , S. Chen , W. Leow , J. Zhang , D. Wang , Y. Lum , Nat. Commun. 2024, 15, 1719. 38409205 10.1038/s41467-024-46175-1 PMC10897157

[9] Y. Zhang , Q. Wu , J. Seow , Y. Jia , X. Ren , Z. Xu , Chem. Soc. Rev. 2024, 53, 8123. 39005214 10.1039/d3cs00913k

[10] X. Ren , T. Wu , Y. Sun , Y. Li , G. Xian , X. Liu , C. Shen , J. Gracia , H.‐J. Gao , H. Yang , Z. Xu , Nat. Commun. 2021, 12, 2608. 33972558 10.1038/s41467-021-22865-y PMC8110536

[11] Z. Du , Z. Meng , X. Gong , Z. Hao , X. Li , H. Sun , X. Hu , S. Yu , H. Tian , Angew. Chem., Int. Ed. 2024, 63, 202317022. 10.1002/anie.202317022 38151463

[12] G. Shen , R. Zhang , L. Pan , F. Hou , Y. Zhao , Z. Shen , W. Mi , C. Shi , Q. Wang , X. Zhang , J.‐J. Zou , Angew. Chem., Int. Ed. 2020, 59, 2313. 10.1002/anie.201913080 31743560

[13] T. Sun , Z. Tang , W. Zang , Z. Li , J. Li , Z. Li , L. Cao , J. Rodriguez , C. Mariano , H. Xu , P. Lyu , X. Hai , H. Lin , X. Sheng , J. Shi , Y. Zheng , Y.‐R. Lu , Q. He , J. Chen , K. Novoselov , C.‐H. Chuang , S. Xi , X. Luo , J. Lu , Nat. Nanotech. 2023, 18, 763. 10.1038/s41565-023-01407-1 37231143

[14] J. Ding , Z. Wei , F. Li , J. Zhang , Q. Zhang , J. Zhou , W. Wang , Y. Liu , Z. Zhang , X. Su , R. Yang , W. Liu , C. Su , H. Yang , Y. Huang , Y. Zhai , B. Liu , Nature Commun 2023, 14, 6550. 37848430 10.1038/s41467-023-42307-1 PMC10582074

[15] X. Kong , Z. Liu , Z. Geng , A. Zhang , Z. Guo , S. Cui , C. Xia , S. Tan , S. Zhou , Z. Wang , J. Zeng , J. Am. Chem. Soc. 2024, 146, 6536. 38412553 10.1021/jacs.3c11088

[16] X. Wang , Y. Fu , D. Tranca , K. Jiang , J. Zhu , J. Zhang , S. Han , C. Ke , C. Lu , X. Zhuang , ACS Appl. Energy Mater. 2021, 4, 2891.

[17] T. Liu , S. Kumar , Chem. Phy. Lett. 2003, 378, 257.

[18] Z. Li , L. Deng , I. Kinloch , R. Young , Prog. in Mater. Sci. 2023, 135, 101089.

[19] K. Li , S. Zhang , X. Zhang , S. Liu , H. Jiang , T. Jiang , C. Chen , Y. Yu , W. Chen , Nano Lett. 2022, 22, 1557. 35104146 10.1021/acs.nanolett.1c04382

[20] H. Liu , L. Tian , Z. Zhang , L. Wang , J. Li , X. Liang , J. Zhuang , H. Yin , D. Yang , G. Zhao , F. Su , D. Wang , Y. Li , J. Am. Chem. Soc. 2024, 146, 20518. 38995120 10.1021/jacs.4c07197

[21] S. Li , Y. Jiao , S. Ding , D. Yang , Z. Niu , G. Li , X. Wang , Y. Luo , J. Mater. Sci. 2022, 57, 17265.

[22] B. Wang , X. Yang , C. Xie , H. Liu , C. Ma , Z. Zhang , Z. Zhuang , A. Han , Z. Zhuang , L. Li , D. Wang , J. Liu , J. Am. Chem. Soc. 2024, 146, 24945. 39214615 10.1021/jacs.4c06173

[23] Y. Zeng , C. Li , B. Li , J. Liang , M. Zachman , D. Cullen , R. Hermann , E. Alp , B. Lavina , S. Karakalos , M. Lucero , B. Zhang , M. Wang , Z. Feng , G. Wang , X. J. , D. Myers , J.‐P. Dodelet , G. Wu , Nat. Catal. 2023, 6, 1215.

[24] S. Liu , C. Li , M. Zachman , Z. Ya , H. Yu , B. Li , M. Wang , J. Braaten , J. Liu , H. Meyer , M. Lucero , A. Kropf , E. Ercan Alp , Q. Gong , Q. Shi , Z. Feng , H. Xu , G. Wang , D. Myers , J. Xie , D. Cullen , S. Litster , G. Wu , Nat. Energy 2022, 7, 652.

[25] Y. Zhou , R. Lu , X. Tao , Z. Qiu , G. Chen , J. Yang , Y. Zhao , X. Feng , K. Müllen , J. Am. Chem. Soc. 2023, 145, 3647. 36744313 10.1021/jacs.2c12933 PMC9936543

[26] O. Lobacheva , M. Chavarha , Y. Yiu , T. Sham , L. Goncharova , J. Appl. Phys. 2014, 116, 013901.

[27] W. Zhu , S. Liu , K. Zhao , Y. Su , Y. Yang , K. Huang , Z. He , Adv. Funct. Mater. 2024, 34, 2402537.

[28] G. Yang , J. Zhu , P. Yuan , Y. Hu , G. Qu , B. Lu , X. Xue , H. Yin , W. Cheng , J. Cheng , W. Xu , J. Li , J. Hu , S. Mu , J. Zhang , Nat. Commun. 2021, 12, 1734. 33741940 10.1038/s41467-021-21919-5 PMC7979714

[29] A. Tanaka , T. Jo , J. Phys. Soc. Jpn. 1994, 63, 2788.

[30] J. Barrio , A. Pedersen , S. Sarma , A. Bagger , M. Gong , S. Favero , C.‐X. Zhao , R. Garcia‐Serres , A. Li , Q. Zhang , F. Jaouen , F. Maillard , A. Kucernak , I. Stephens , M.‐M. Titrici , Adv. Mater. 2023, 35, 2211022. 10.1002/adma.202211022 36739474

[31] Y. Gu , X. Wang , M. Humayun , L. Li , H. Sun , X. Xu , X. Xue , A. Habibi‐Yangjeh , K. Temst , C. Wang , Chin. J. Catal. 2022, 43, 839.

[32] M. Drosou , C. Mitsopoulou , D. Pantazis , Polyhedron 2021, 208,115399.

[33] S. Saha , P. Chandra , S. Mandal , Physica. B 2022, 642, 414128.

[34] G. Kresse , J. Furthmüller , Comp. Mater. Sci. 1996, 6, 15.

[35] G. Kresse , J. Hafner , Phys. Rev. B 1994, 49, 14251. 10.1103/physrevb.49.14251 10010505

[36] G. Kresse , D. Joubert , Phys. Rev. B 1999, 59, 1758.

[37] P. Blöchl , O. Jepsen , O. Andersen , Phys. Rev. B 1994, 49, 16223. 10.1103/physrevb.49.16223 10010769

[38] J. Klimes , D. Bowler , A. Michaelides , Phys. Rev. B 2011, 83, 195131.

[39] J. Norskov , T. Bligaard , A. Logadottir , J. Kitchin , J. Chen , S. Pandelov , U. Stimming , J. Electrochem. Soc. 2005, 152, J23.

[40] G. Henkelman , A. Arnaldsson , H. Jónsson , Comp. Mater. Sci. 2006, 36, 354.

