# Supplementary material for: Tuning Transition Metal 3d Spin state on Single‐atom Catalysts for Selective Electrochemical CO2 Reduction
Source: Adv Mater. 2025 Mar 13;37(16):2417034. doi: 10.1002/adma.202417034 (PMC12016740; doi:10.1002/adma.202417034)
Supplement: Supplementary file 1 — Supporting Information [file ADMA-37-2417034-s001.pdf]

ADVANCED MATERIALS

Supporting Information

for *Adv. Mater.*, DOI 10.1002/adma.202417034

Tuning Transition Metal 3d Spin state on Single-atom Catalysts for Selective Electrochemical CO_2 Reduction

Yipeng Zang, Yan Liu, Ruihu Lu, Qin Yang, Bingqing Wang, Mingsheng Zhang, Yu Mao, Ziyun Wang and Yanwei Lum**

Supporting Information

Tuning transition metal 3*d* spin state on single-atom catalysts for selective electrochemical CO_2 reduction

Yipeng Zang[1,4], Yan Liu[2,4], Ruihu Lu[2], Qin Yang[1], Bingqing Wang[1], Mingsheng Zhang[3], Yu Mao[2], Ziyun Wang[2]* and Yanwei Lum[1,3]*

[1]Department of Chemical and Biomolecular Engineering, National University of Singapore, Singapore 117585, Republic of Singapore

[2]School of Chemical Sciences, University of Auckland, Auckland 1010, New Zealand

[3]Institute of Materials Research and Engineering (IMRE), Agency for Science, Technology and Research (A*STAR), 2 Fusionopolis Way, Innovis #08-03, Singapore, 138634, Republic of Singapore

[4]These authors contributed equally to this work.

*Corresponding author: ziyun.wang@auckland.ac.nz

*Corresponding author: lumyw@nus.edu.sg

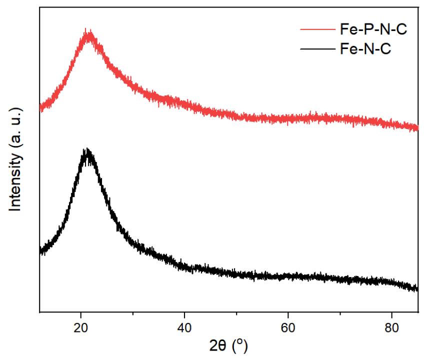

Figure S1. XRD patterns of Fe-P-N-C and Fe-N-C.

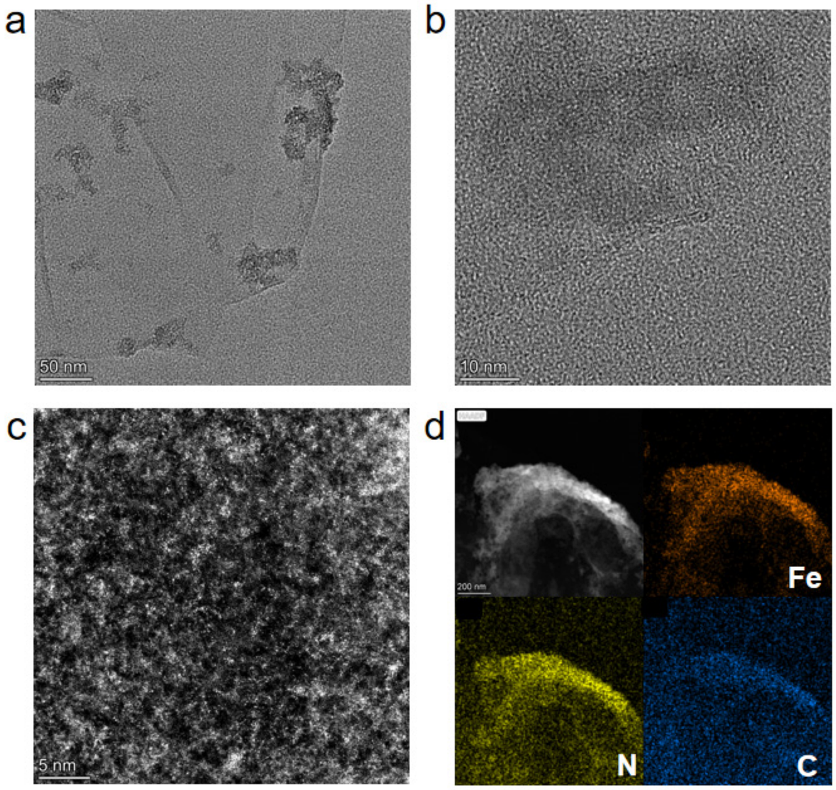

Figure S2. (a) TEM and (b) HRTEM images. (c) AC-ADF-STEM image. (d) HAADF-STEM image and EDS elemental mapping of Fe-N-C sample.

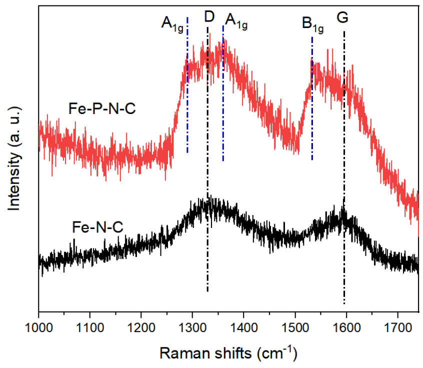

Figure S3. Raman spectra of Fe-P-N-C and Fe-N-C.

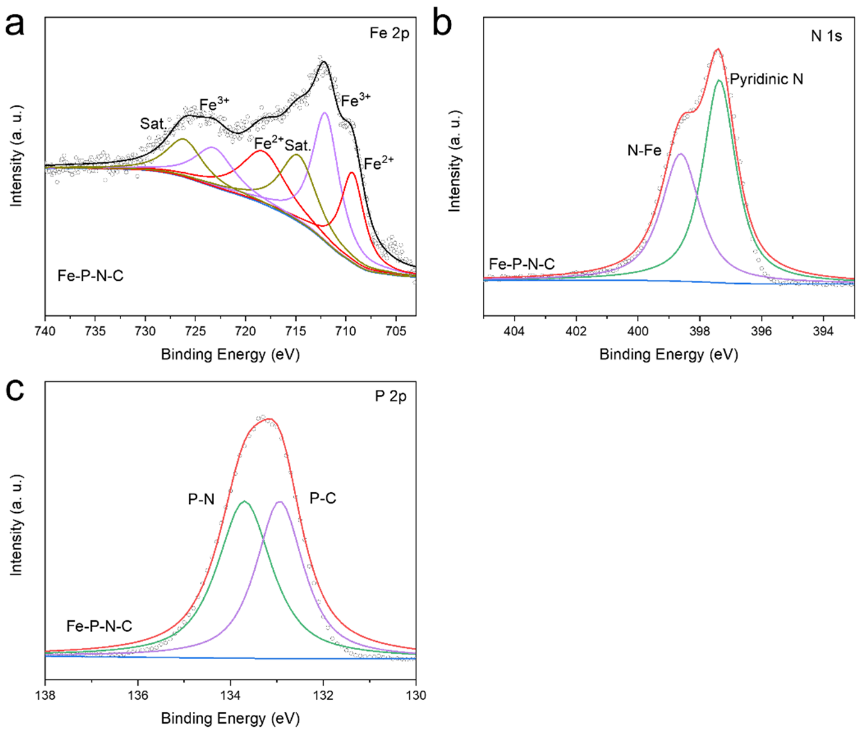

Figure S4. XPS spectra of Fe-P-N-C. (a) Fe 2p. (b) N 1s. (c) P 2p.

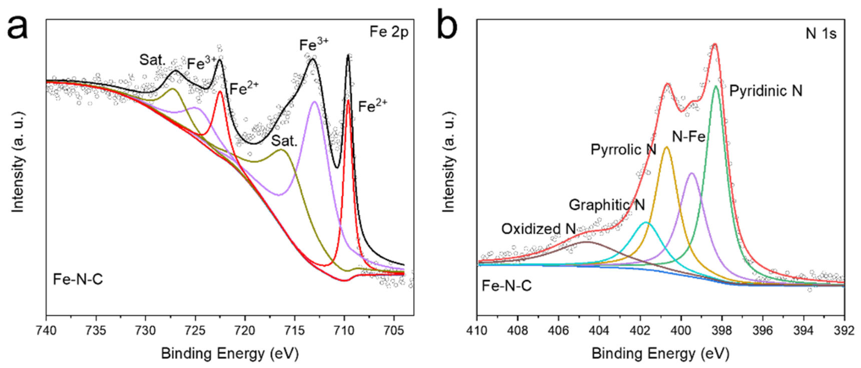

Figure S5. XPS spectra of Fe-N-C. (a) Fe 2p. (b) N 1s.

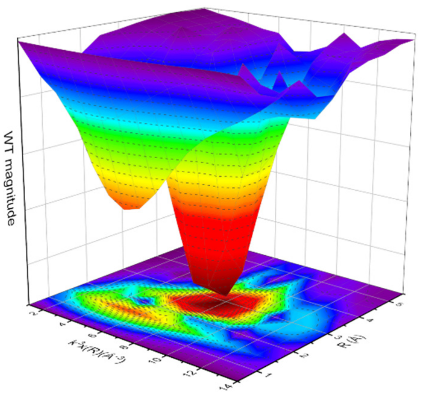

Figure S6. WT spectrum of Fe_2O_3.

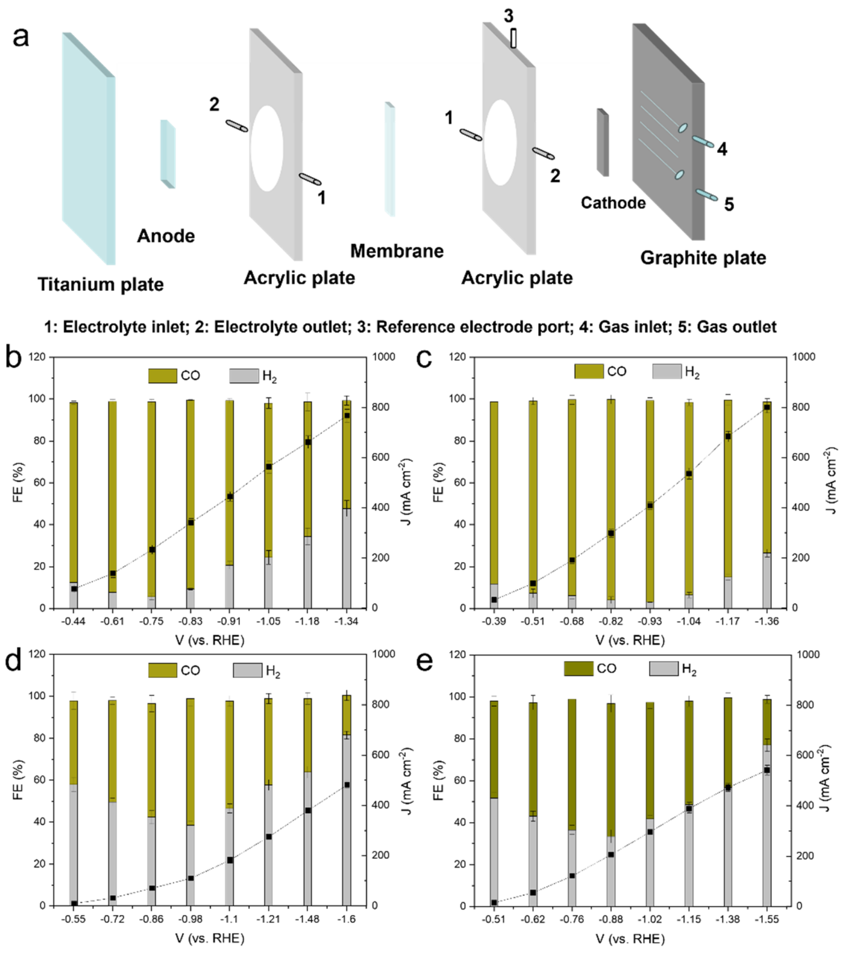

Figure S7. (a) The schematic of the flow cell system used for CO_2R tests. Potential-dependent Faradaic efficiency of each product and total current density over (b) Fe-N-C, (c) Fe-P-N-C (d) N-C and (e) P-N-C.

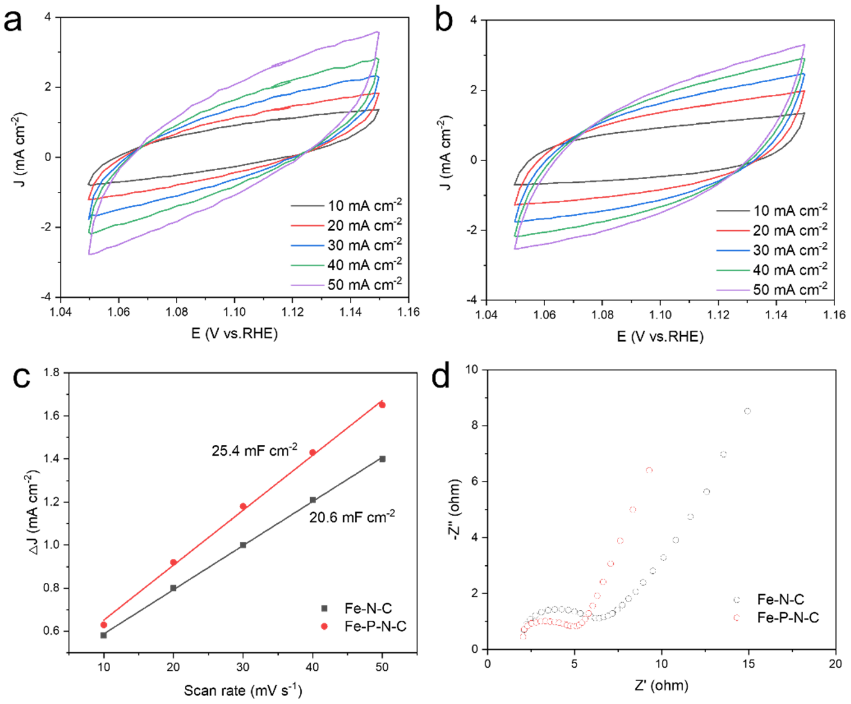

Figure S8. Cyclic voltammograms of (a) Fe-N-C and (b) Fe-P-N-C. (c) Electrochemical double-layer capacitances of Fe-N-C and Fe-P-N-C. (d) Electrochemical impedance spectroscopy of the Fe-N-C and Fe-P-N-C electrodes recorded at -1.0 V vs. RHE.

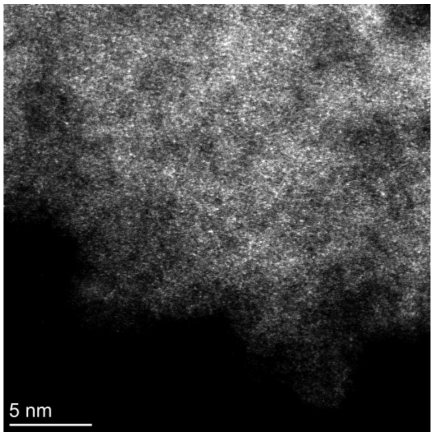

Figure S9. AC-ADF-STEM image of Fe-P-N-C after CO_2R.

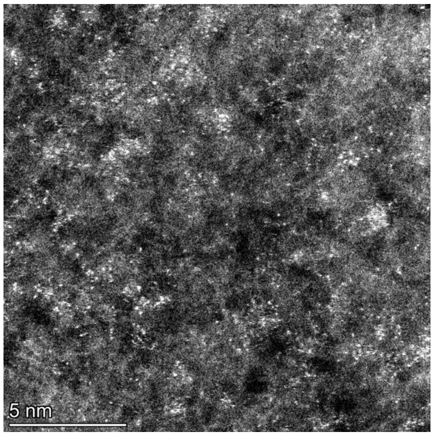

Figure S10. AC-ADF-STEM image of Fe-N-C after CO_2R.

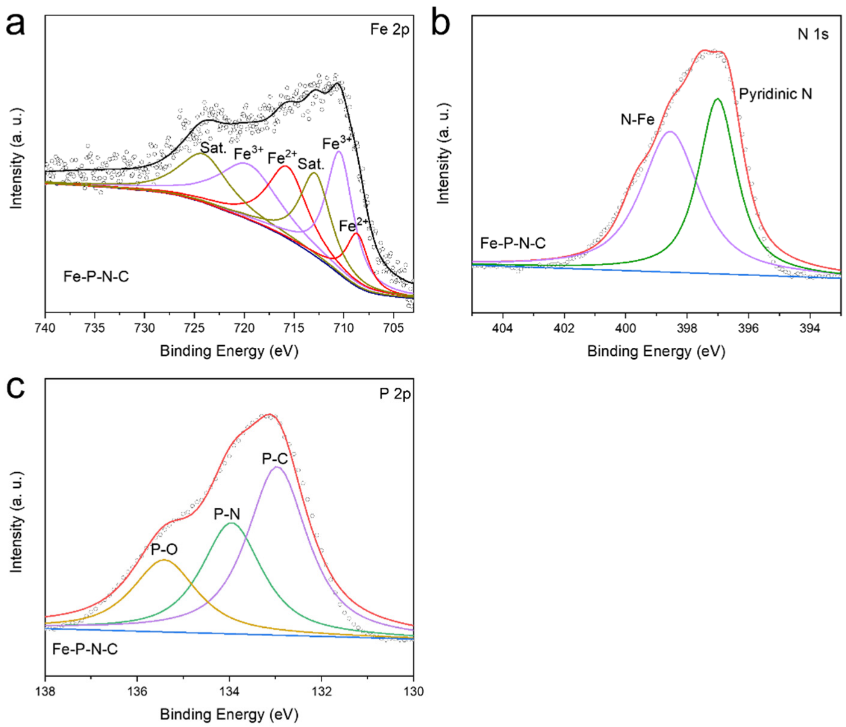

Figure S11. XPS spectra of Fe-P-N-C after CO_2R. (a) Fe 2p. (b) N 1s. (c) P 2p.

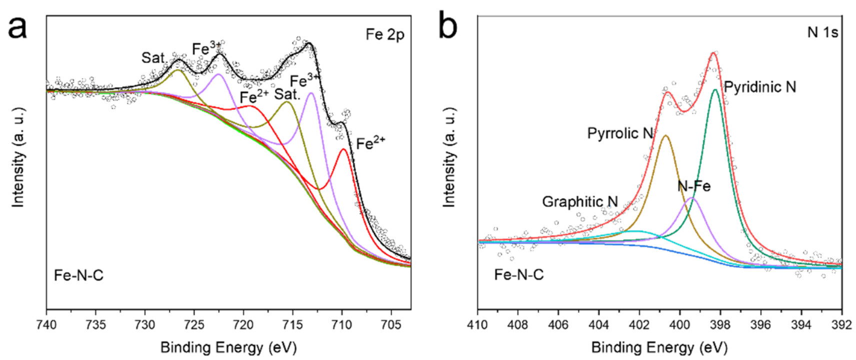

Figure S12. XPS spectra of Fe-N-C after CO_2R. (a) Fe 2p. (b) N 1s.

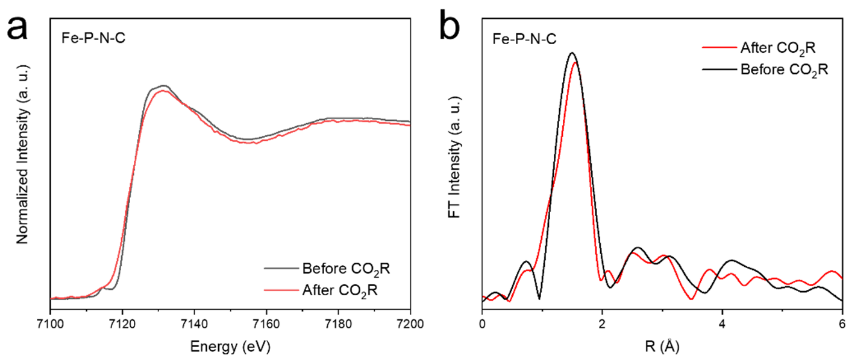

Figure S13. (a) XANES spectra and (b) FT R space spectra of Fe-P-N-C before and after CO_2R.

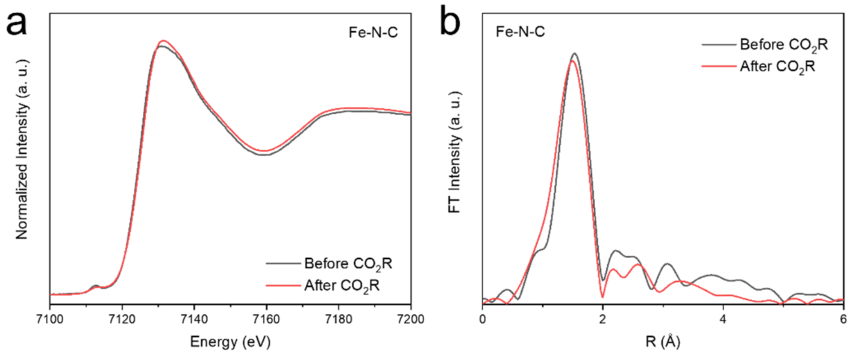

Figure S14. (a) XANES spectra and (b) FT R space spectra of Fe-N-C before and after CO_2R.

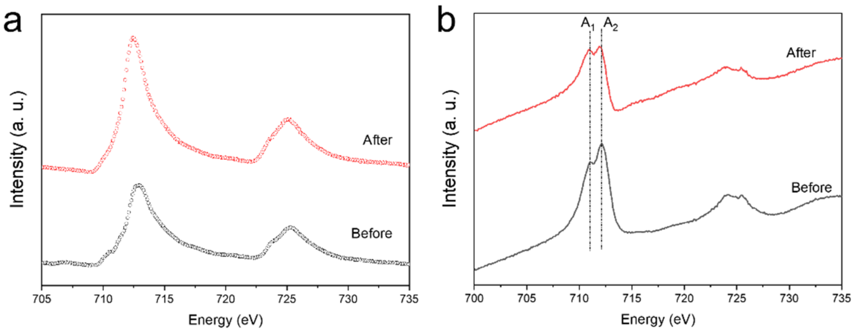

Figure S15. Fe L-edge XAS spectra of (a) Fe-N-C and (b) Fe-P-N-C before and after CO_2R test.

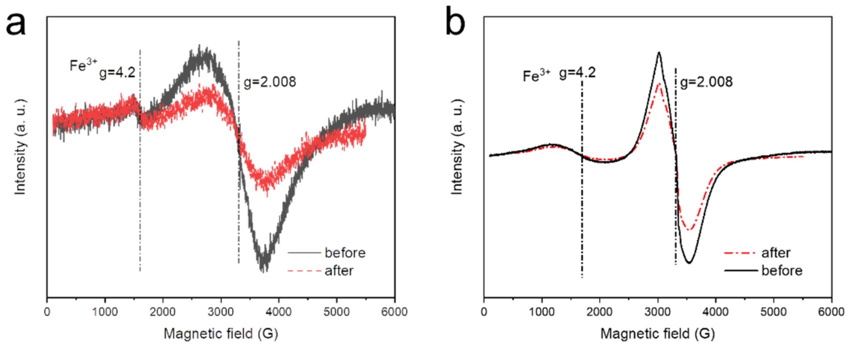

Figure S16. Electron Spin Resonance spectra of (a) Fe-N-C and (b) Fe-P-N-C before and after CO_2R test.

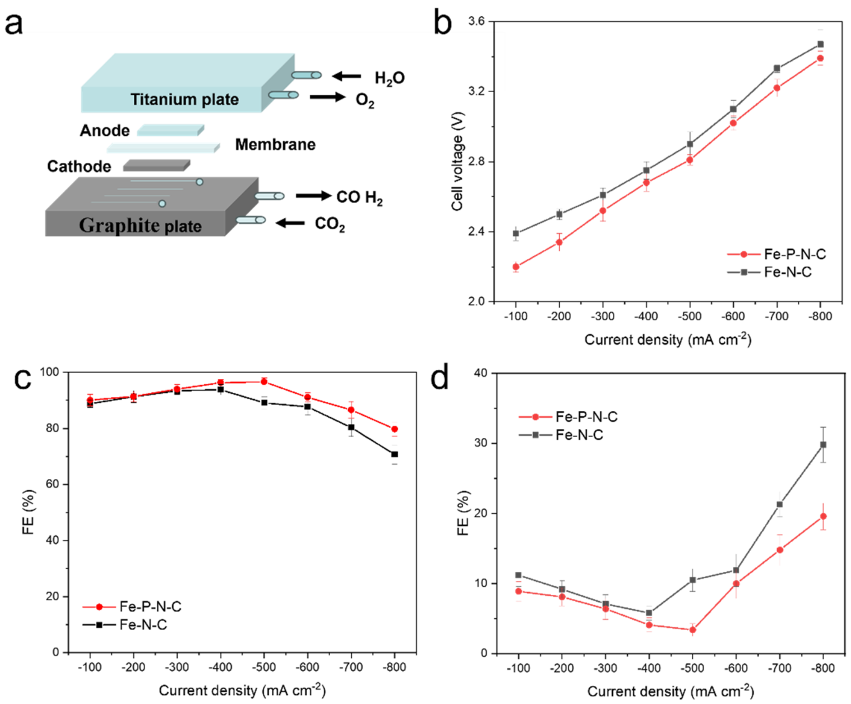

Figure S17. (a) The schematic of the MEA system used for CO_2R tests. (b) Current density-dependent cell voltage. Current density-dependent Faradaic efficiency of (c) CO and (d) H_2 over Fe-P-N-C and Fe-N-C electrodes .

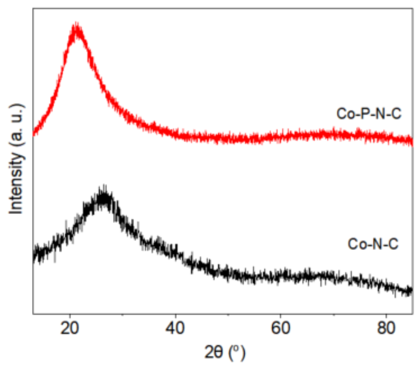

Figure S18. XRD patterns of Co-P-N-C and Co-N-C.

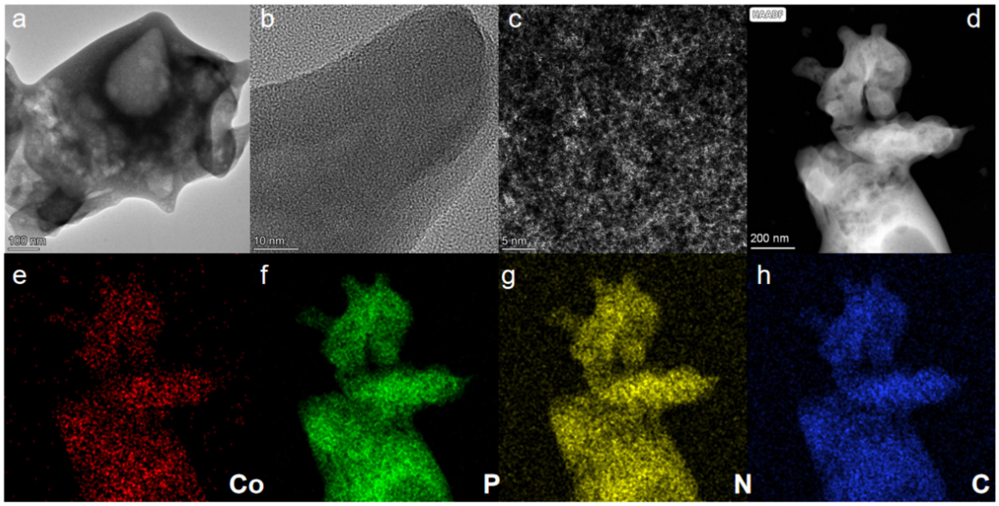

Figure S19. (a) TEM and (b) HRTEM images. (c) AC-ADF-STEM image. (d) HAADF-STEM image and (e-h) EDX elemental mapping of Co-P-N-C sample.

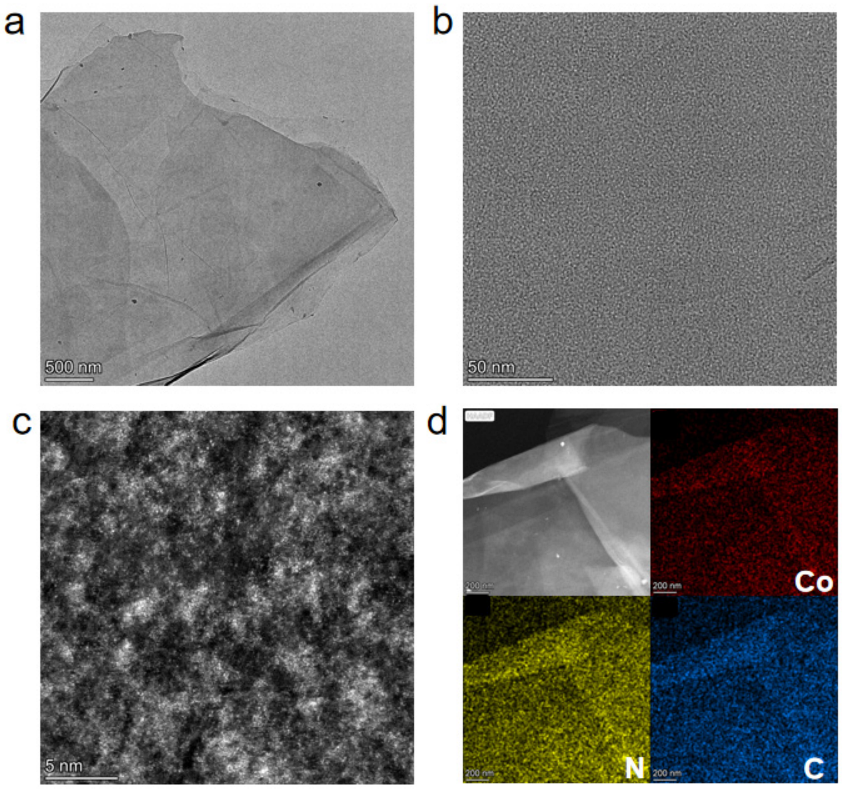

Figure S20. (a) TEM and (b) HRTEM images. (c) AC-ADF-STEM image. (d) HAADF-STEM image and EDX elemental mapping of Co-N-C sample.

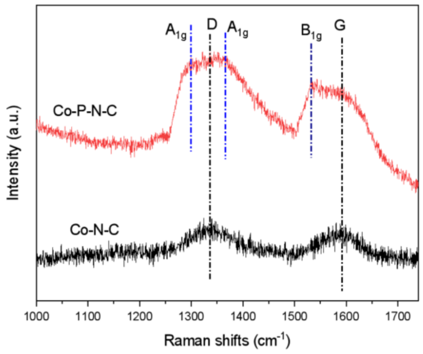

Figure S21. Raman spectra of Co-P-N-C and Co-N-C.

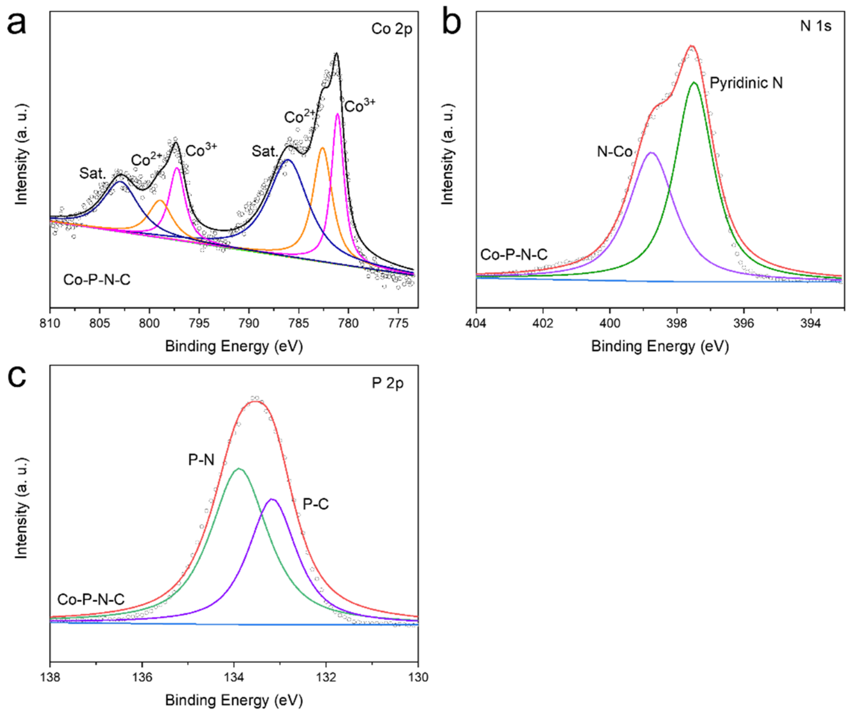

Figure S22. XPS spectra of Co-P-N-C. (a) Co 2p. (b) N 1s. (c) P 2p.

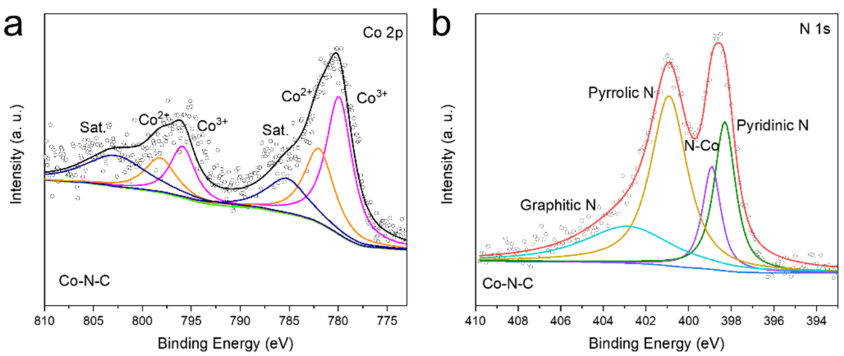

Figure S23. XPS spectra of Co-N-C. (a) Co 2p. (b) N 1s.

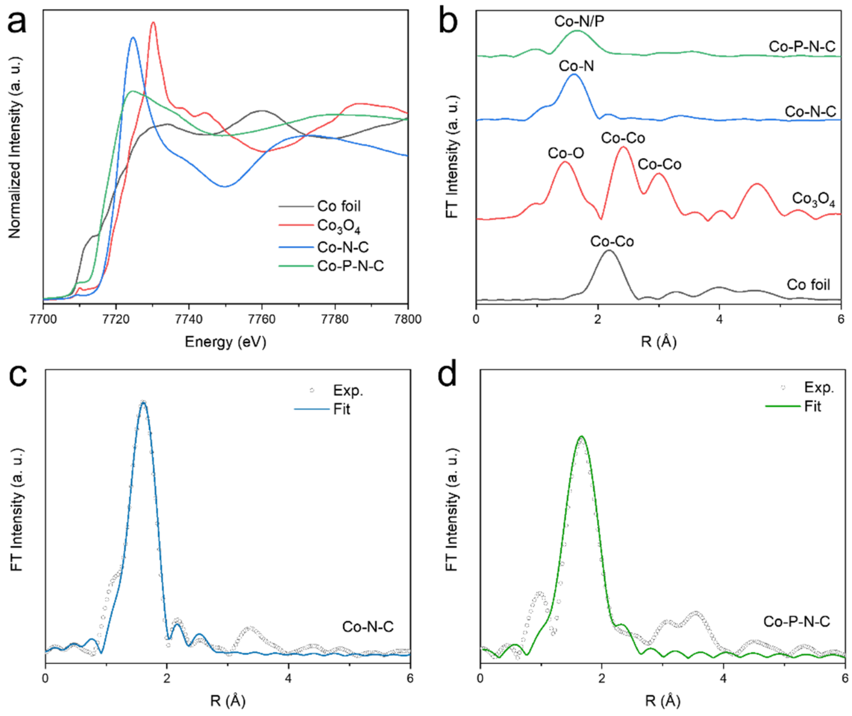

Figure S24. (a) XANES spectra and (b) the corresponding FT R space spectra of Co foil, Co_3O_4, Co-P-N-C and Co-N-C. R space and fitting spectra of (c) Co-N-C and (d) Co-P-N-C.

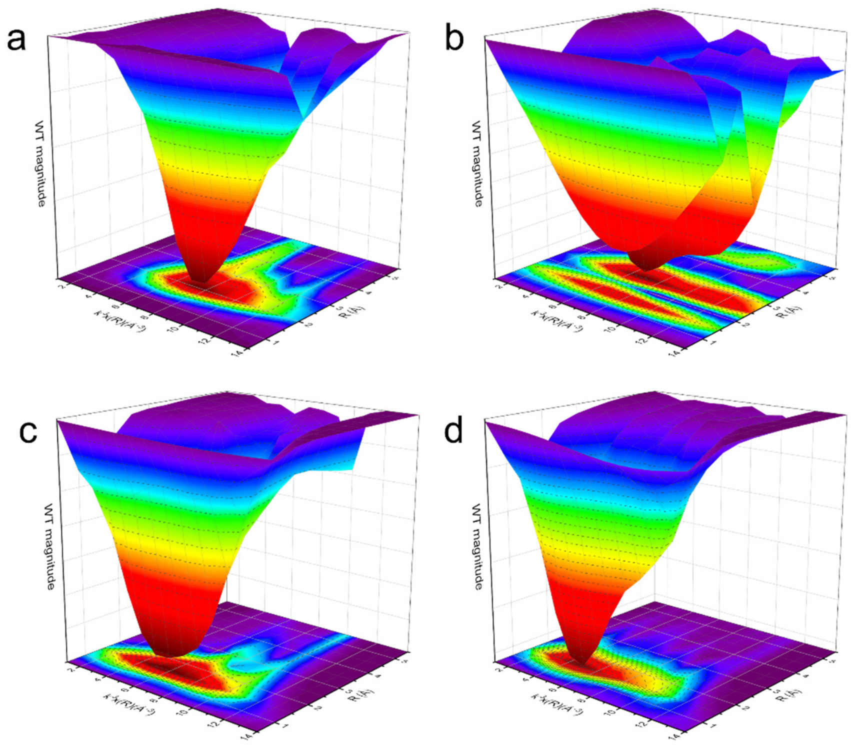

Figure S25. WT spectra of (a) Co foil, (b) Co_3O_4, (c) Co-P-N-C and (d) Co-N-C.

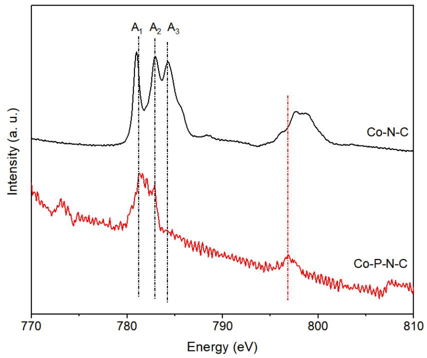

Figure S26. Co L edge XAS of Co-P-N-C and Co-N-C.

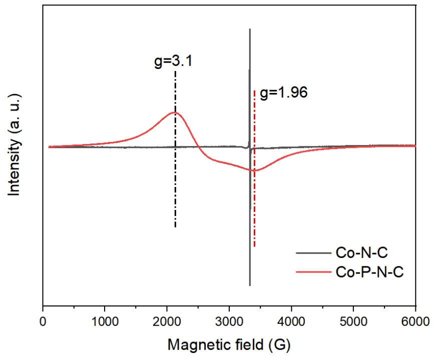

Figure S27. ESR spectra of Co-P-N-C and Co-N-C.

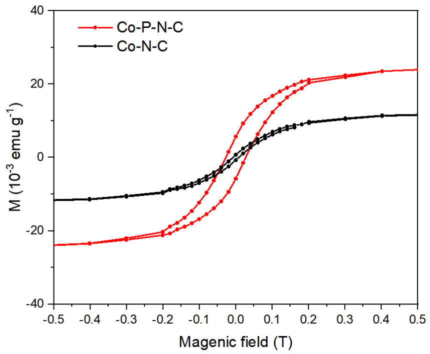

Figure S28. M-H curves of Co-P-N-C and Co-N-C.

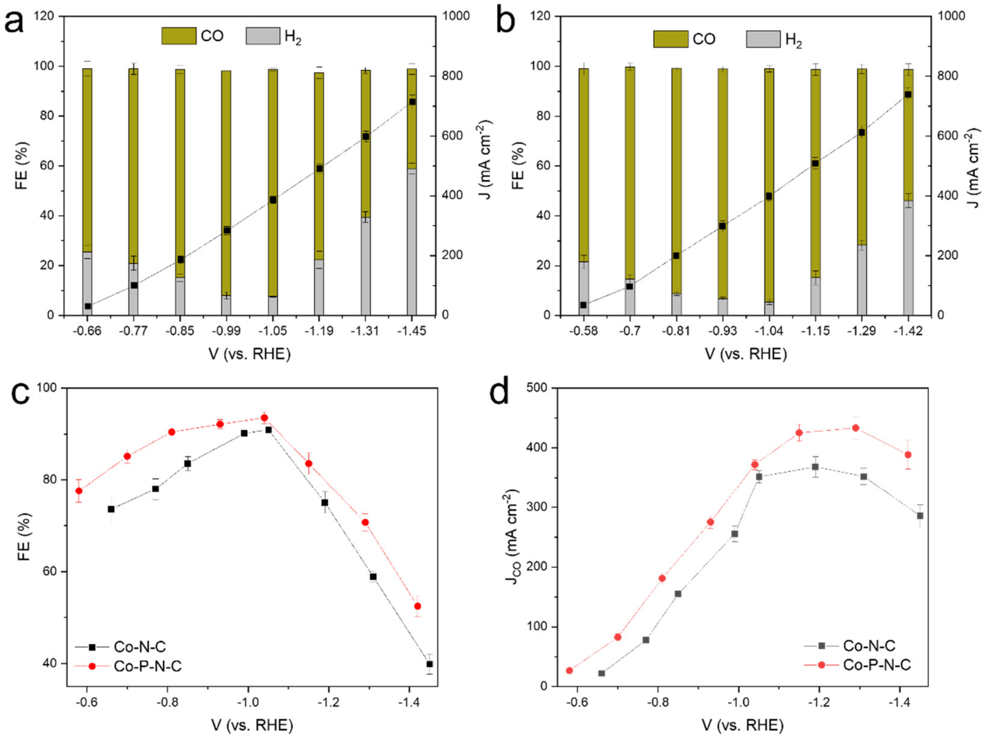

Figure S29. Potential-dependent Faradaic efficiency of each product and total current density over (a) Co-N-C and (b) Co-P-N-C. (c) CO FEs and (d) partial current densities of Co-P-N-C and Co-N-C.

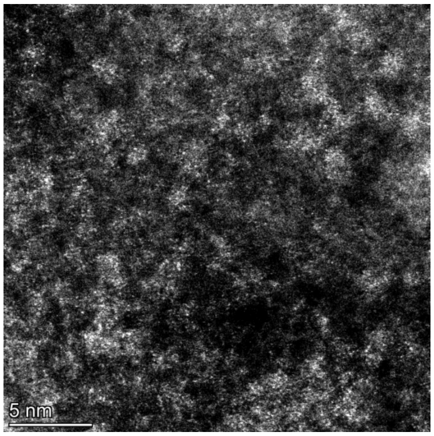

Figure S30. AC-ADF-STEM image of Co-P-N-C after CO_2R.

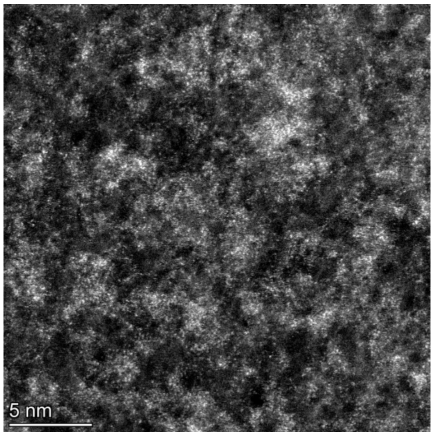

Figure S31. AC-ADF-STEM image of Co-N-C after CO_2R.

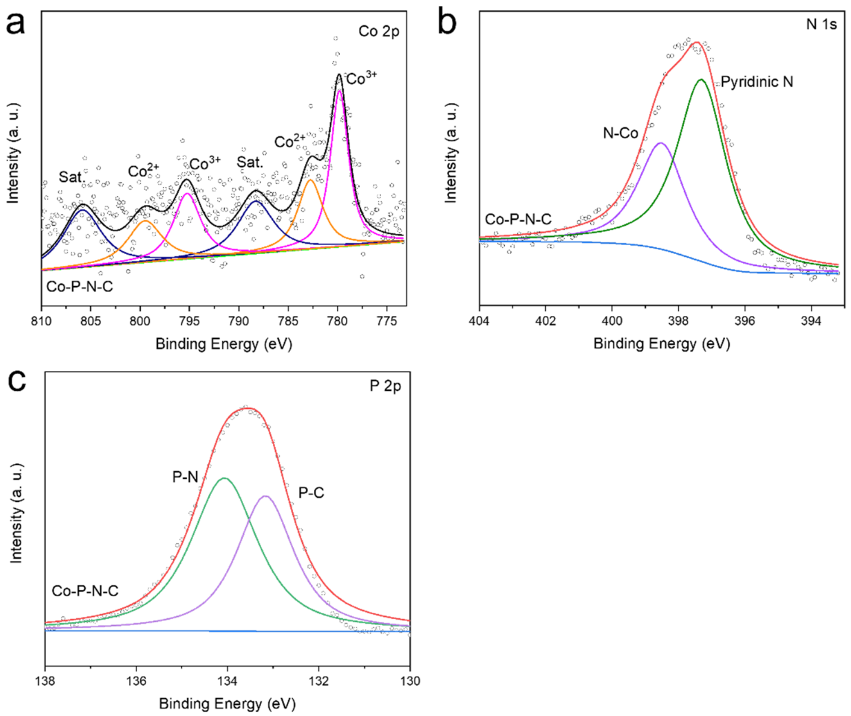

Figure S32. XPS spectra of Co-P-N-C after CO_2R. (a) Co 2p. (b) N 1s. (c) P 2p.

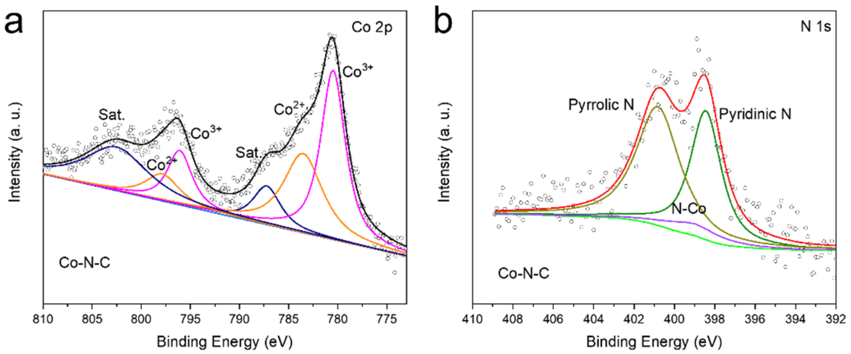

Figure S33. XPS spectra of Co-N-C after CO_2R. (a) Co 2p. (b) N 1s.

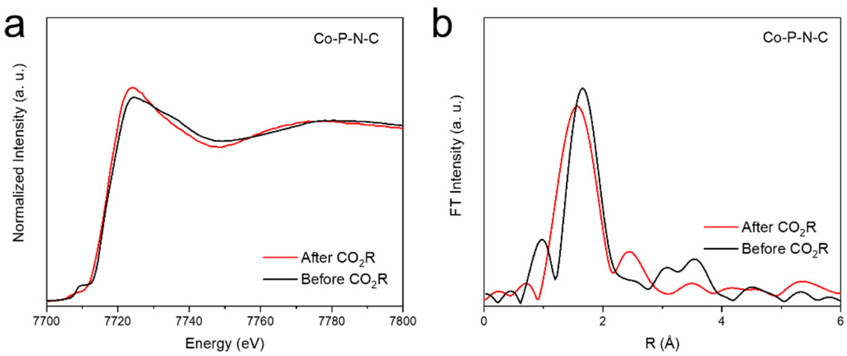

Figure S34. (a) XANES spectra and (b) FT R space spectra of Co-P-N-C before and after CO_2R.

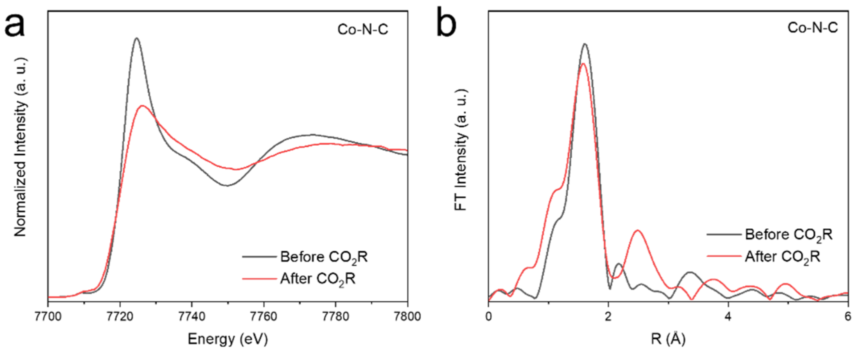

Figure S35. (a) XANES spectra and (b) FT R space spectra of Co-N-C before and after CO_2R.

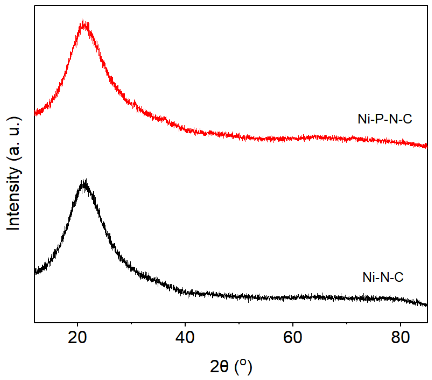

Figure S36. XRD patterns of Ni-P-N-C and Ni-N-C.

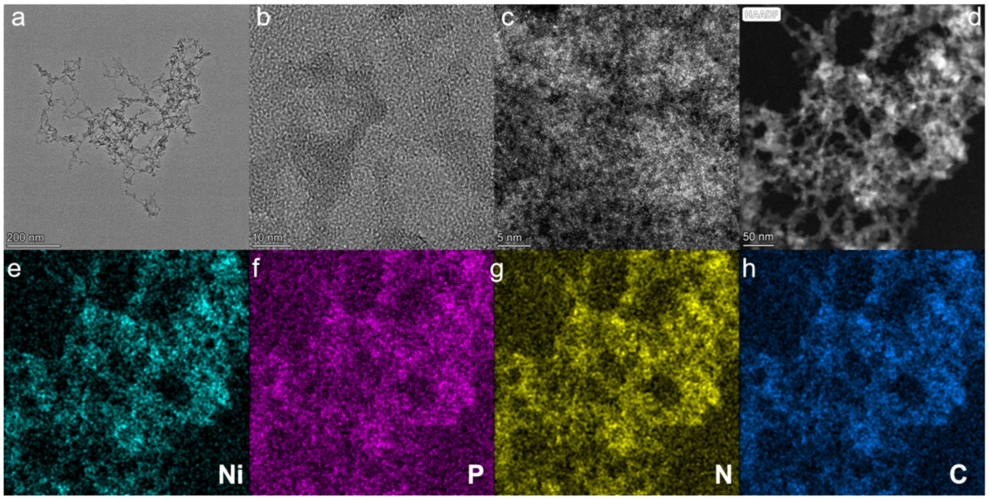

Figure S37. (a) TEM and (b) HRTEM images. (c) AC-ADF-STEM image. (d) HAADF-STEM image and (e-h) EDX elemental mapping of Ni-P-N-C sample.

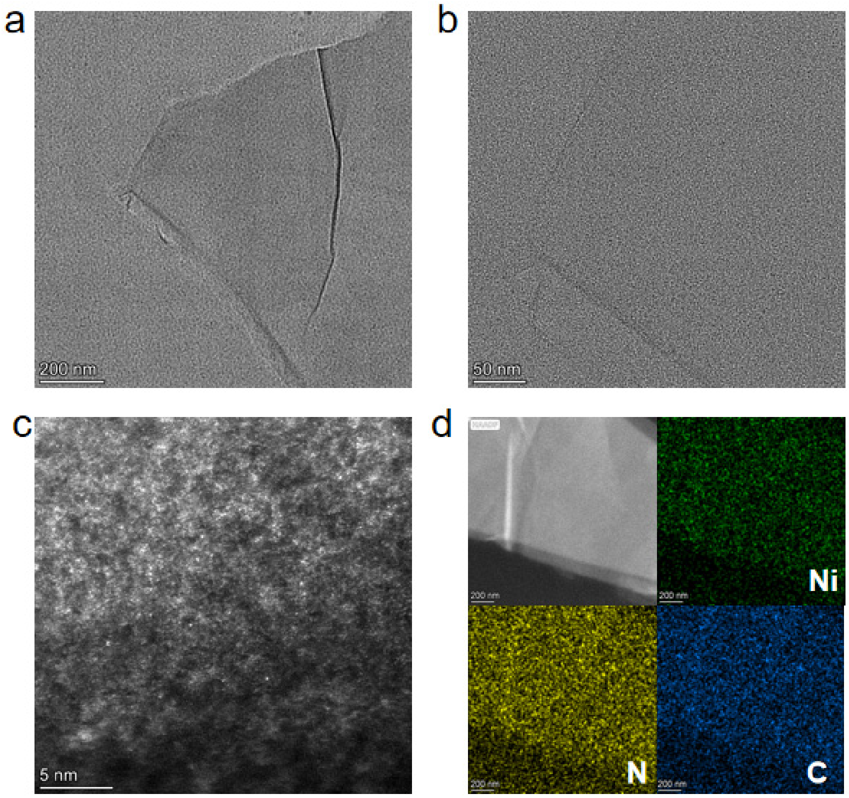

Figure S38. (a) TEM and (b) HRTEM images. (c) AC-ADF-STEM image. (d) HAADF-STEM image and EDX elemental mapping of Ni-N-C sample.

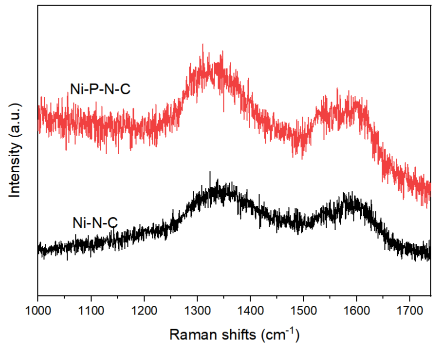

Figure S39. Raman spectra of Ni-P-N-C and Ni-N-C.

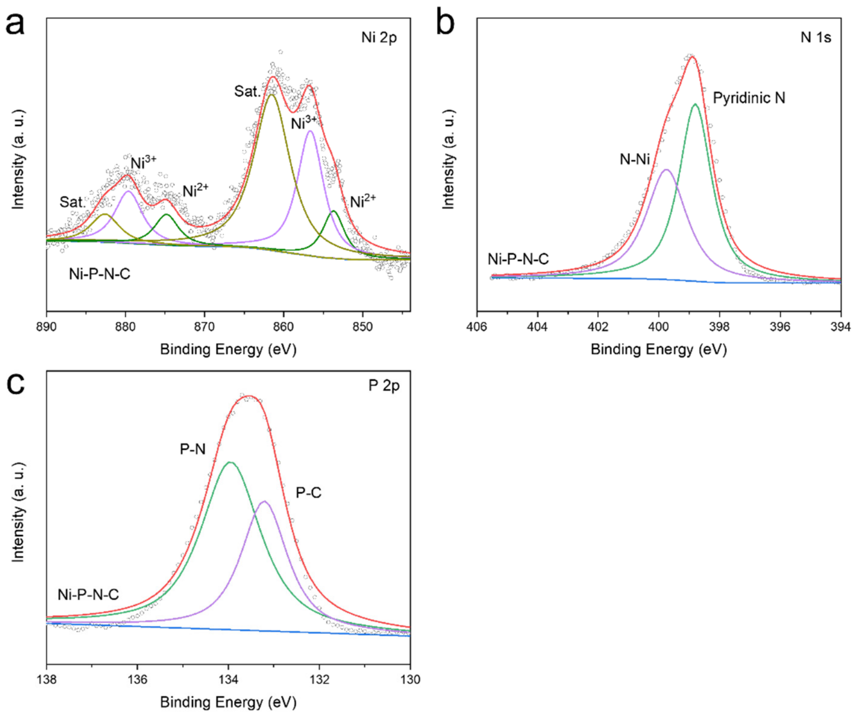

Figure S40. XPS spectra of Ni-P-N-C. (a) Ni 2p. (b) N 1s. (c) P 2p.

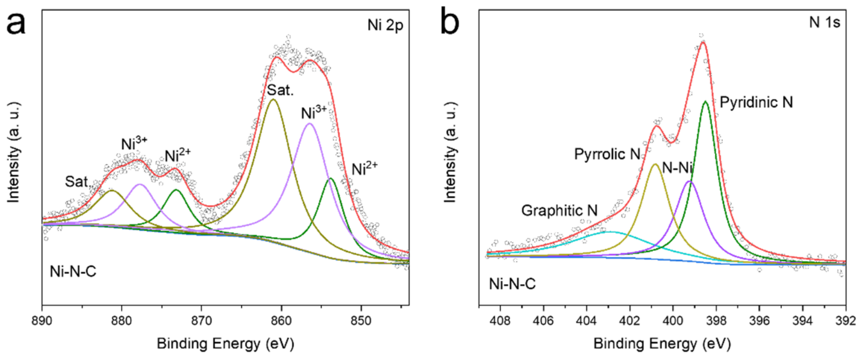

Figure S41. XPS spectra of Ni-N-C. (a) Ni 2p. (b) N 1s.

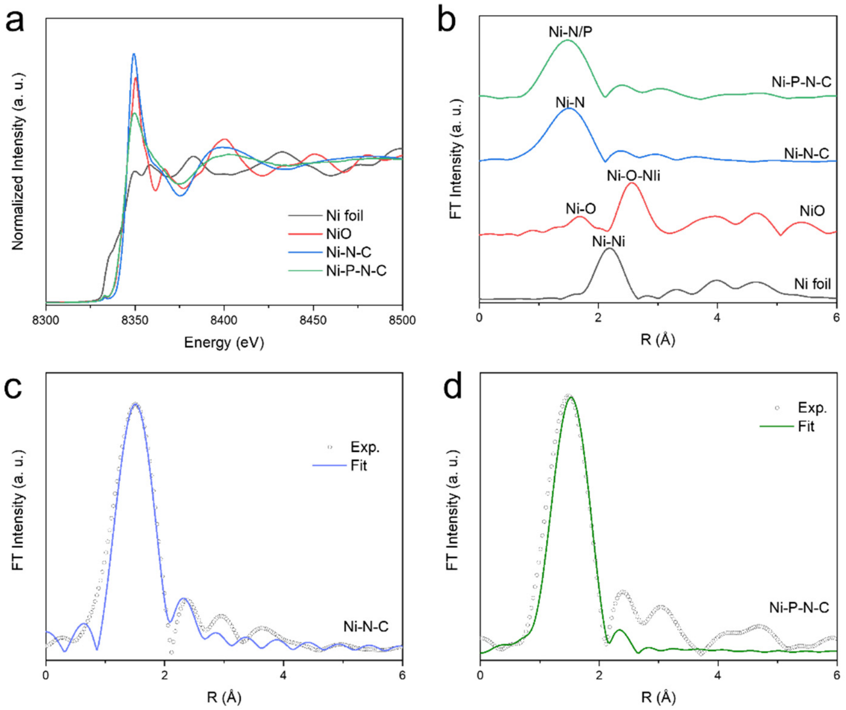

Figure S42. (a) XANES spectra and (b) the corresponding FT R space spectra of Ni foil, NiO, Ni-P-N-C and Ni-N-C. FT R space and fitting spectra of (c) Ni-N-C and (d) Ni-P-N-C.

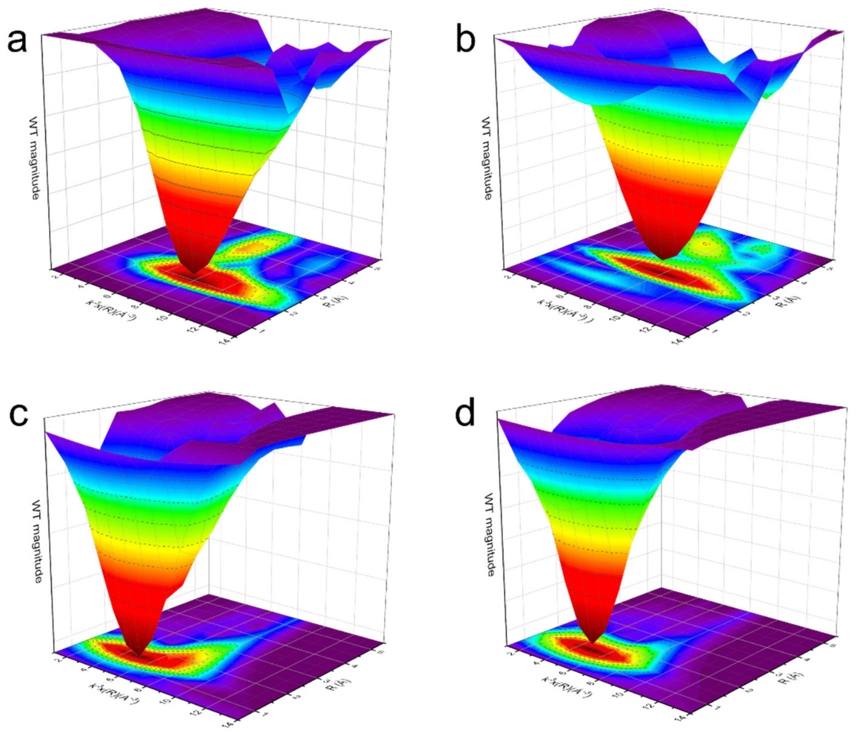

Figure S43. WT spectra of (a) Ni foil, (b) NiO, (c) Ni-P-N-C and (d) Ni-N-C.

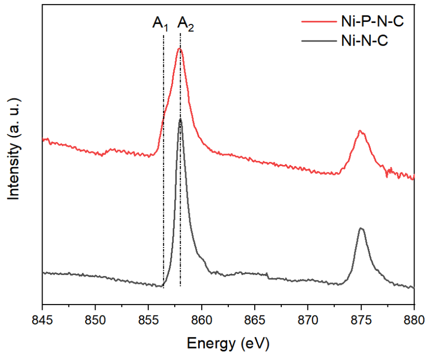

Figure S44. Ni L edge XAS of Ni-P-N-C and Ni-N-C.

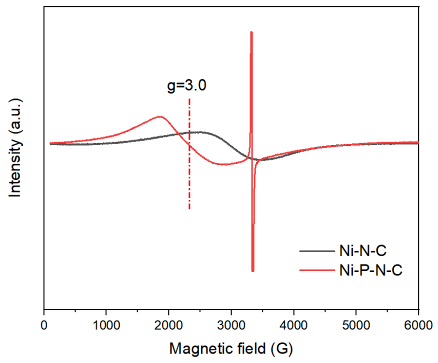

Figure S45. ESR spectra of Ni-P-N-C and Ni-N-C.

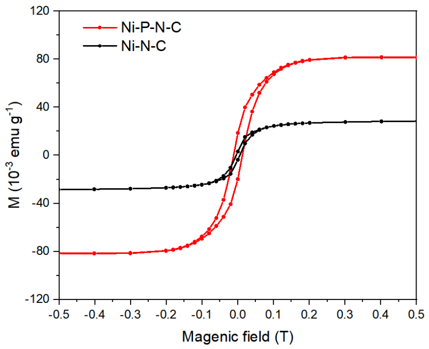

Figure S46. M-H curves of Ni-P-N-C and Ni-N-C.

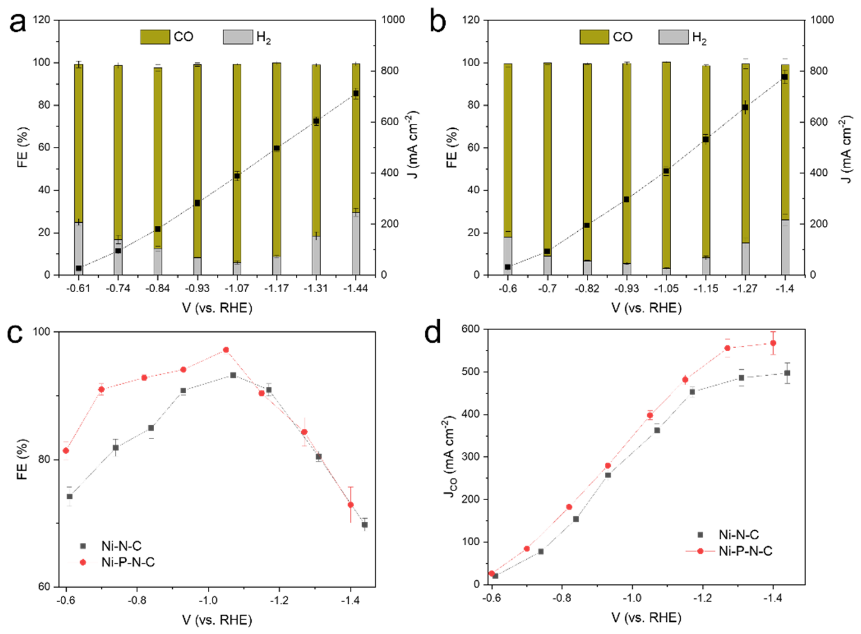

Figure S47. Potential-dependent Faradaic efficiency of each product and total current density over (a) Ni-N-C and (b) Ni-P-N-C. (c) CO FEs and (d) partial current densities of Ni-P-N-C and Ni-N-C.

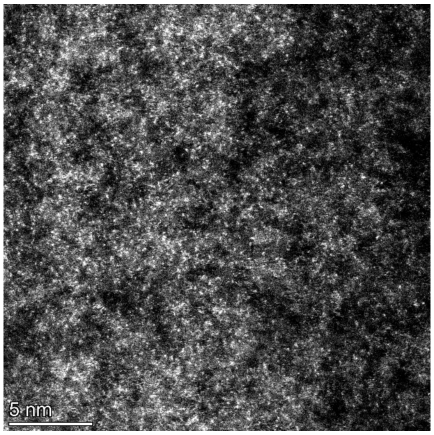

Figure S48. AC-ADF-STEM image of Ni-P-N-C after CO_2R.

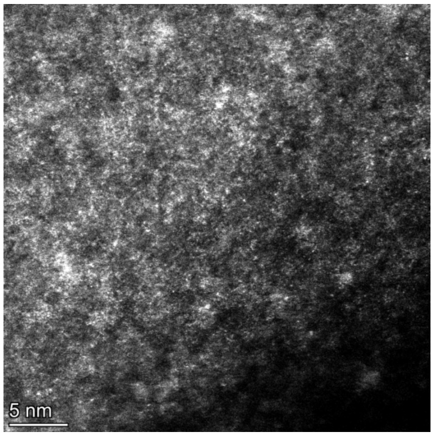

Figure S49. AC-ADF-STEM image of Ni-N-C after CO_2R.

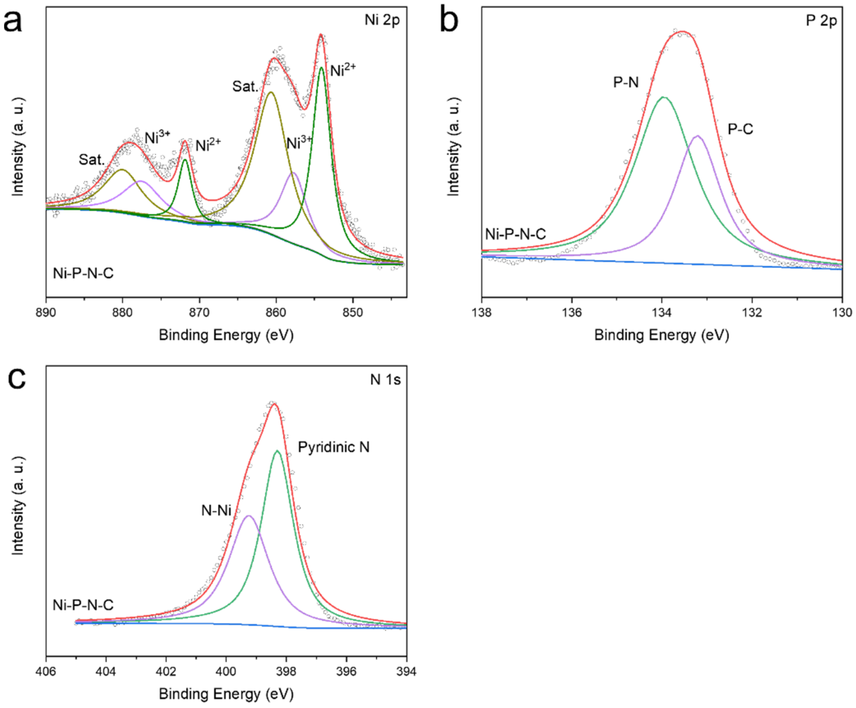

Figure S50. XPS spectra of Ni-P-N-C after CO_2R. (a) Ni 2p. (b) P 2p. (c) N 1s.

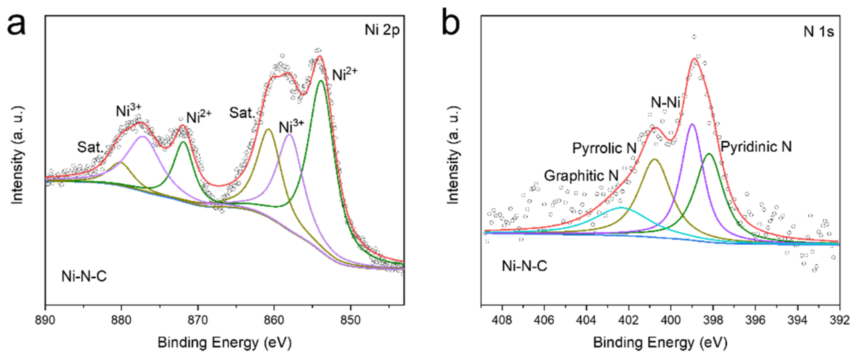

Figure S51. XPS spectra of Ni-N-C after CO_2R. (a) Ni 2p. (b) N 1s.

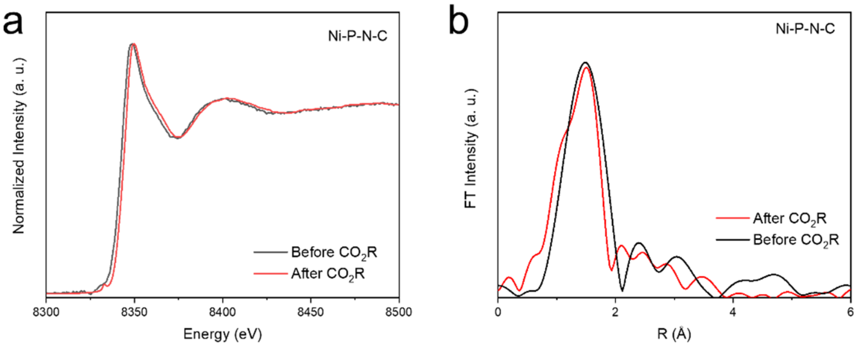

Figure S52. (a) XANES spectra and (b) FT R space spectra of Ni-P-N-C before and after CO_2R.

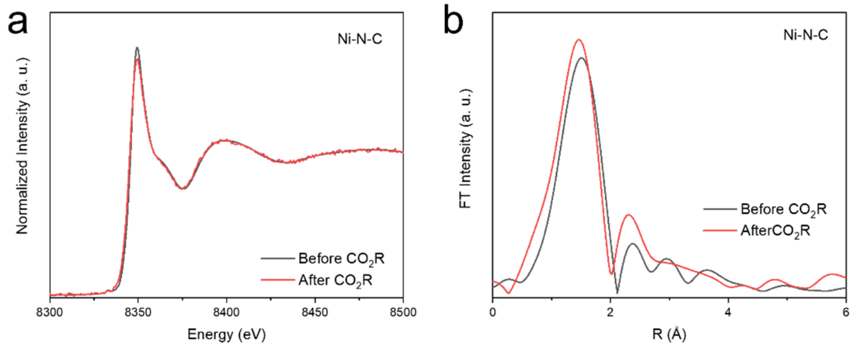

Figure S53. (a) XANES spectra and (b) FT R space spectra of Ni-N-C before and after CO_2R.

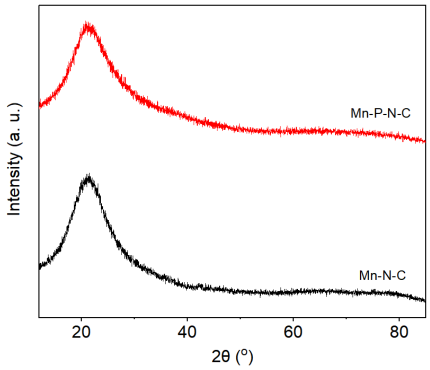

Figure S54. XRD patterns of Mn-P-N-C and Mn-N-C.

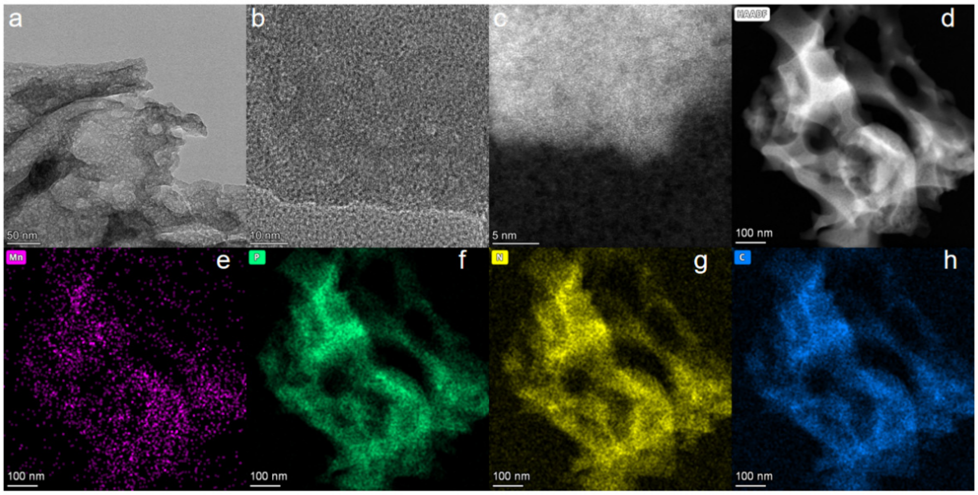

Figure S55. (a) TEM and (b) HRTEM images. (c) AC-ADF-STEM image. (d) HAADF-STEM image and (e-h) EDX elemental mapping of Mn-P-N-C sample.

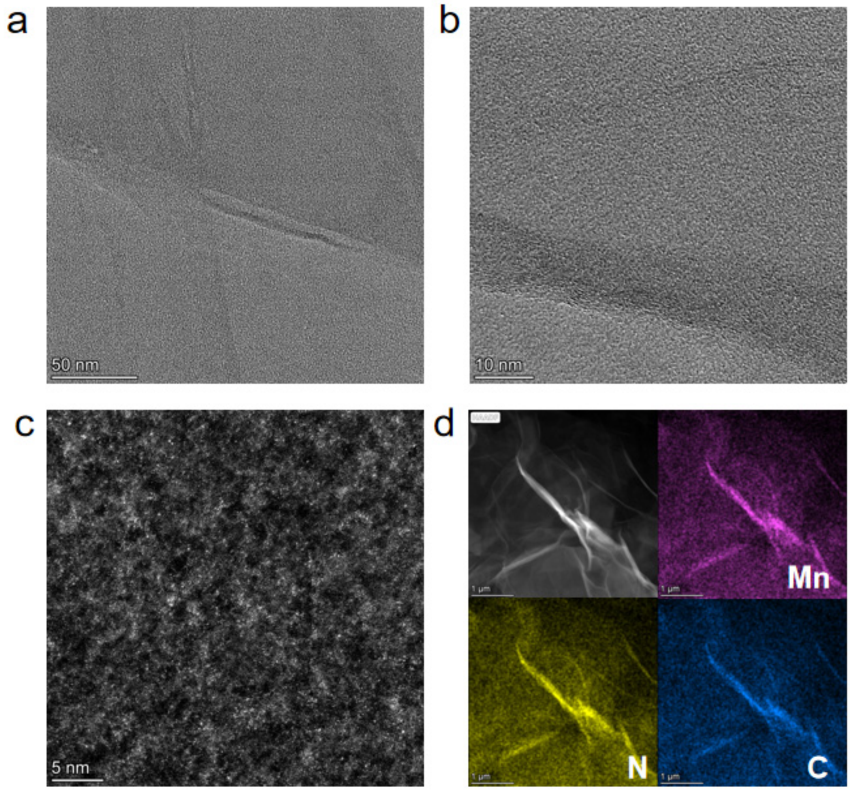

Figure S56. (a) TEM and (b) HRTEM images. (c) AC-ADF-STEM image. (d) HAADF-STEM image and EDX elemental mapping of Mn-N-C sample.

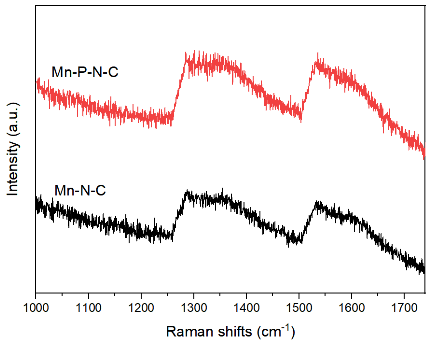

Figure S57. Raman spectra of Mn-P-N-C and Mn-N-C.

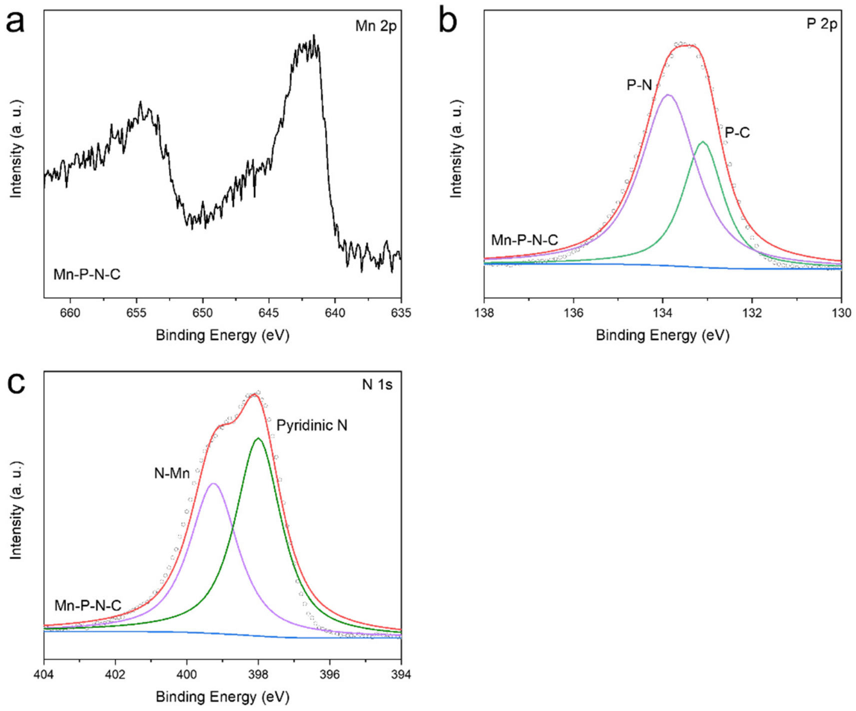

Figure S58. XPS spectra of Mn-P-N-C. (a) Mn 2p. (b) P 2p. (c) N 1s.

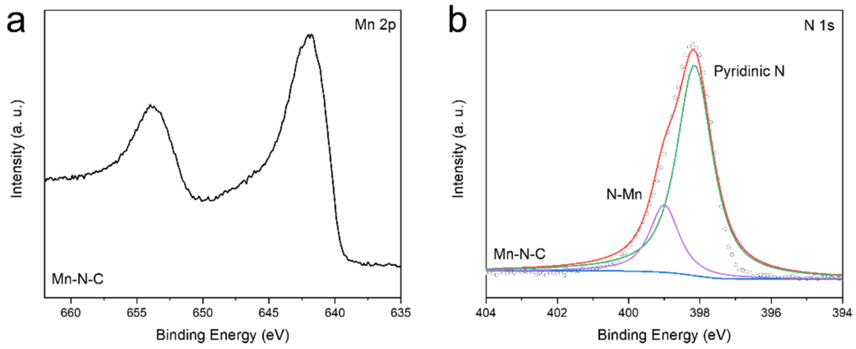

Figure S59. XPS spectra of Mn-N-C. (a) Mn 2p. (b) N 1s.

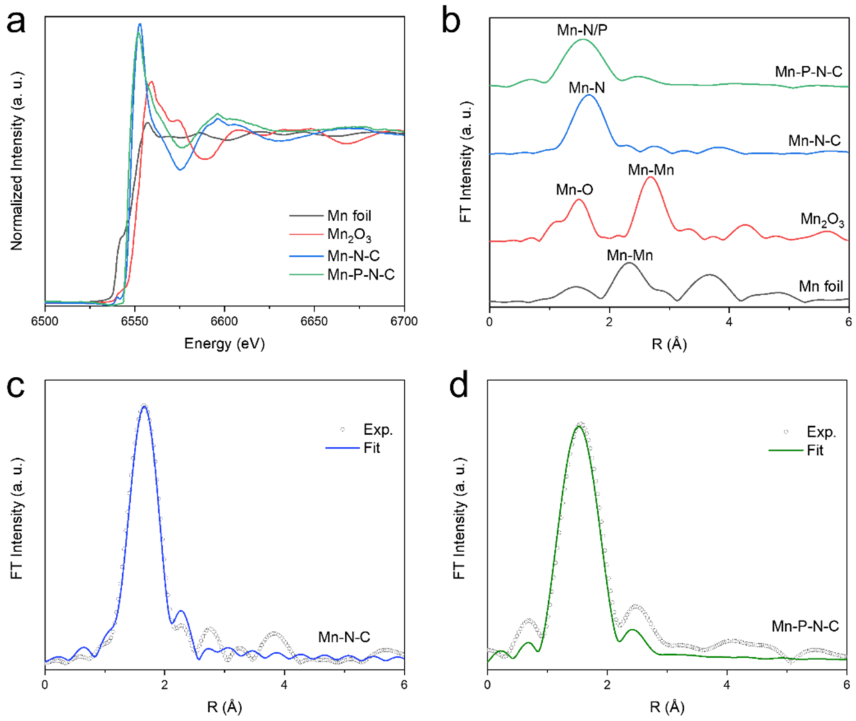

Figure S60. (a) XANES spectra and (b) the corresponding FT R space of Mn foil, Mn_2O_3, Mn-P-N-C and Mn-N-C. FT R space and fitting spectra of (c) Mn-N-C and (d) Mn-P-N-C.

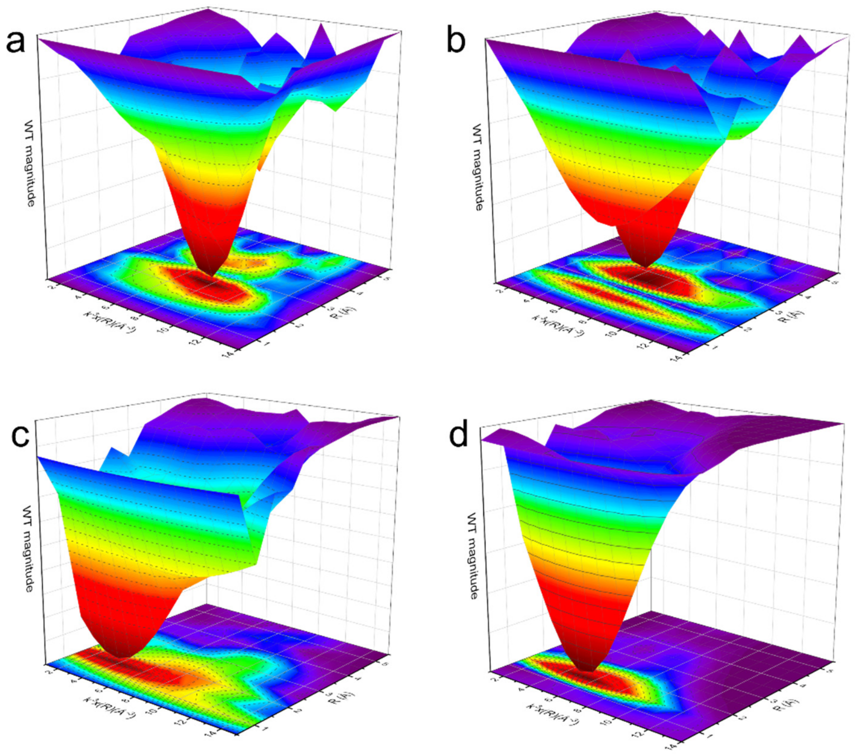

Figure S61. WT spectra of (a) Mn foil, (b) Mn_2O_3, (c) Mn-P-N-C and (d) Mn-N-C.

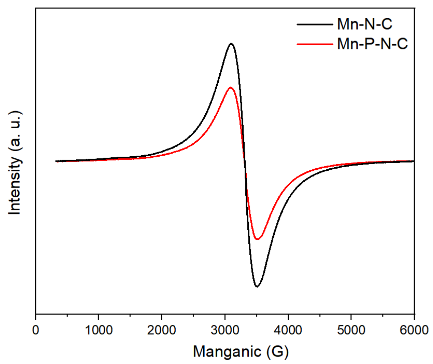

Figure S62. ESR spectra of Mn-P-N-C and Mn-N-C.

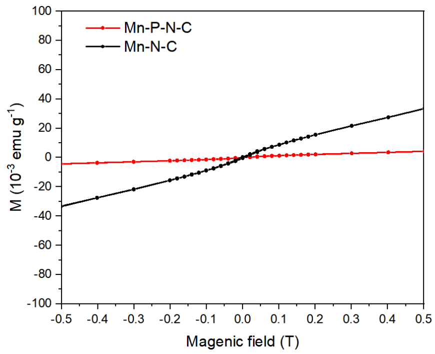

Figure S63. M-H curves of Mn-P-N-C and Mn-N-C.

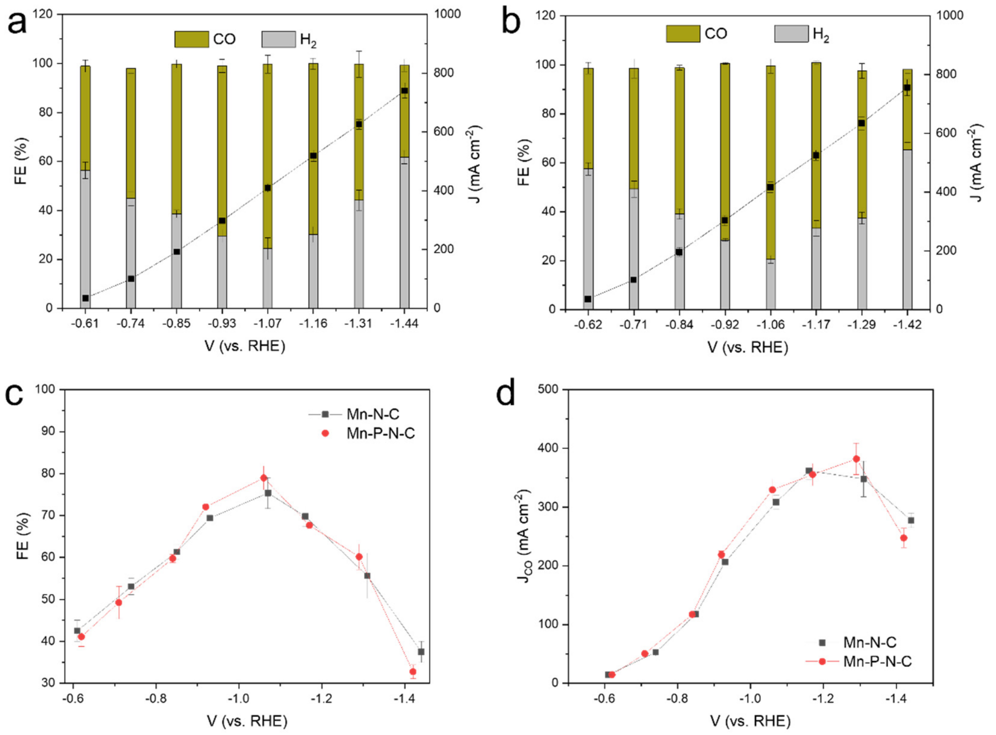

Figure S64. Potential-dependent Faradaic efficiency of each product and total current density over (a) Mn-N-C and (b) Mn-P-N-C. (c) CO FEs and (d) partial current densities of Mn-P-N-C and Mn-N-C.

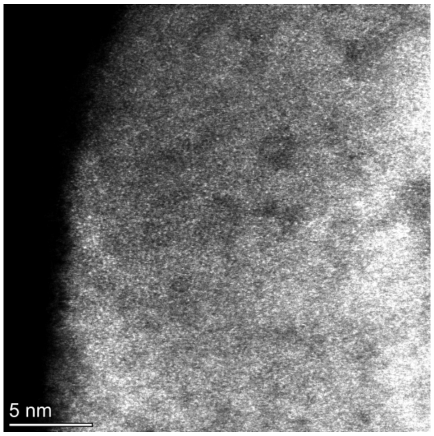

Figure S65. AC-ADF-STEM image of Mn-P-N-C after CO_2R.

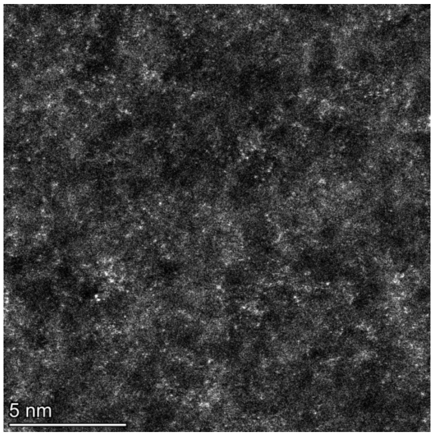

Figure S66. AC-ADF-STEM image of Mn-N-C after CO_2R.

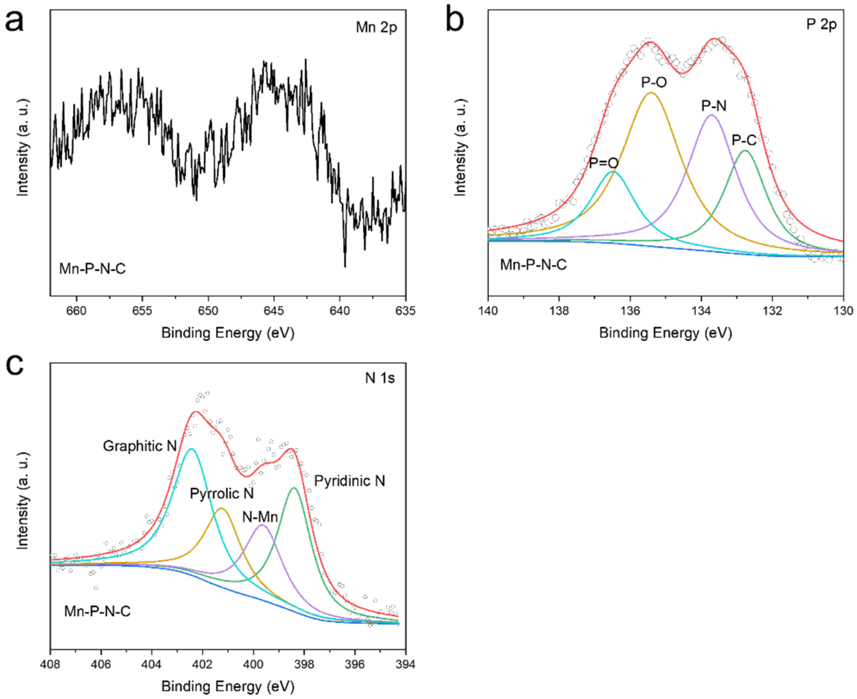

Figure S67. XPS spectra of Mn-P-N-C after CO_2R. (a) Mn 2p. (b) P 2p. (c) N 1s.

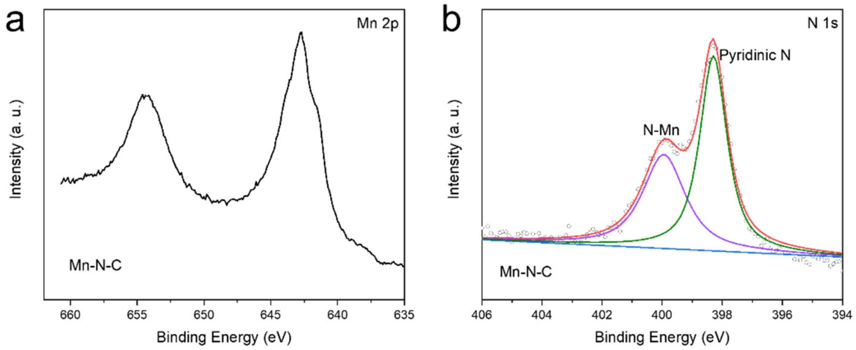

Figure S68. XPS spectra of Mn-N-C after CO_2R. (a) Mn 2p. (b) N 1s.

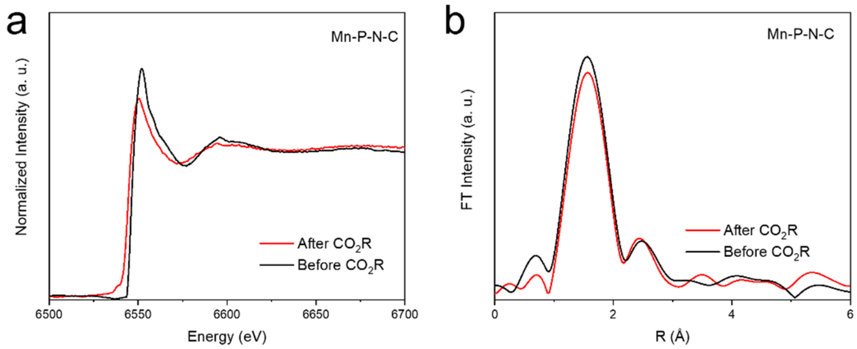

Figure S69. (a) XANES spectra and (b) FT R space spectra of Mn-P-N-C before and after CO_2R.

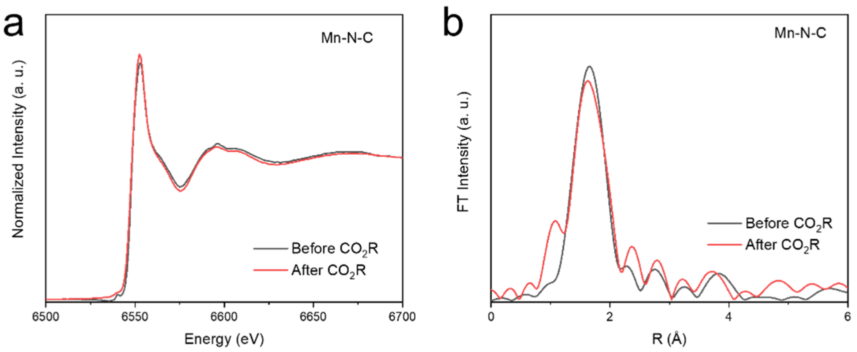

Figure S70. (a) XANES spectra and (b) FT R space spectra of Mn-N-C before and after CO_2R.

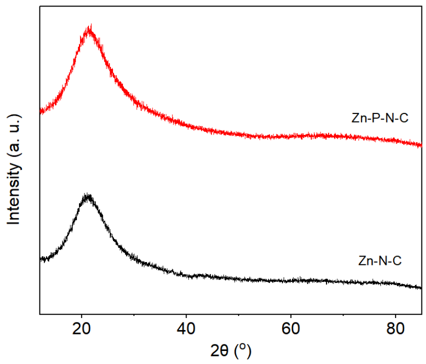

Figure S71. XRD patterns of Zn-P-N-C and Zn-N-C.

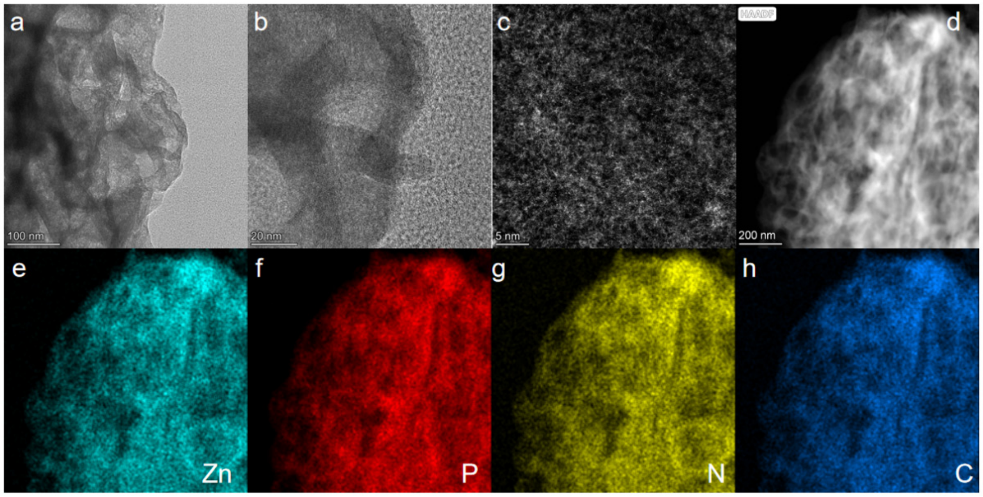

Figure S72. (a) TEM and (b) HRTEM images. (c) AC-ADF-STEM image. (d) HAADF-STEM image and (e-h) EDX elemental mapping of Zn-P-N-C sample.

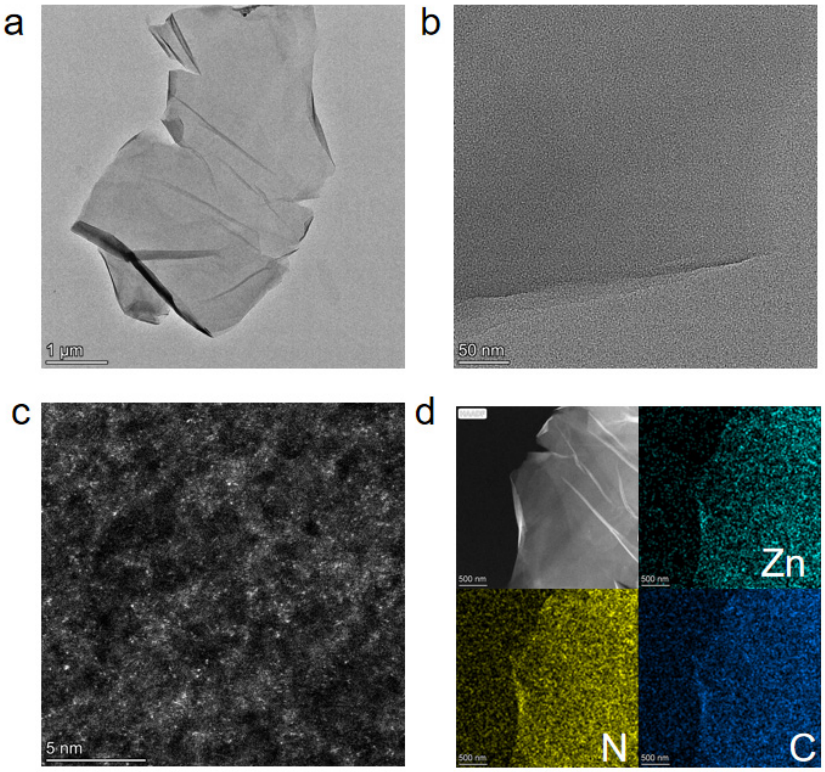

Figure S73. (a) TEM and (b) HRTEM images. (c) AC-ADF-STEM image. (d) HAADF-STEM image and EDX elemental mapping of Zn-N-C sample.

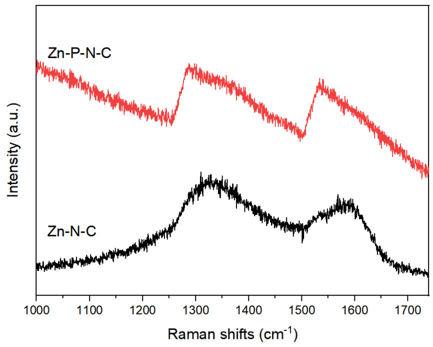

Figure S74. Raman spectra of Zn-P-N-C and Zn-N-C.

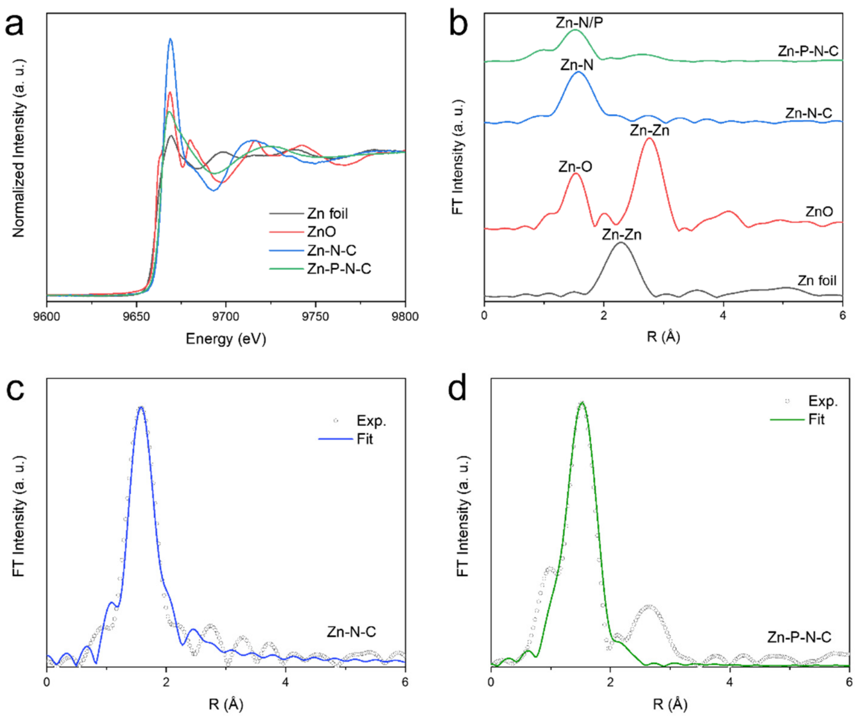

Figure S75. (a) XANES spectra and (b) the corresponding FT R space of Zn foil, ZnO, Zn-P-N-C and Zn-N-C. FT R space and fitting spectra of (c) Zn-N-C and (d) Zn-P-N-C.

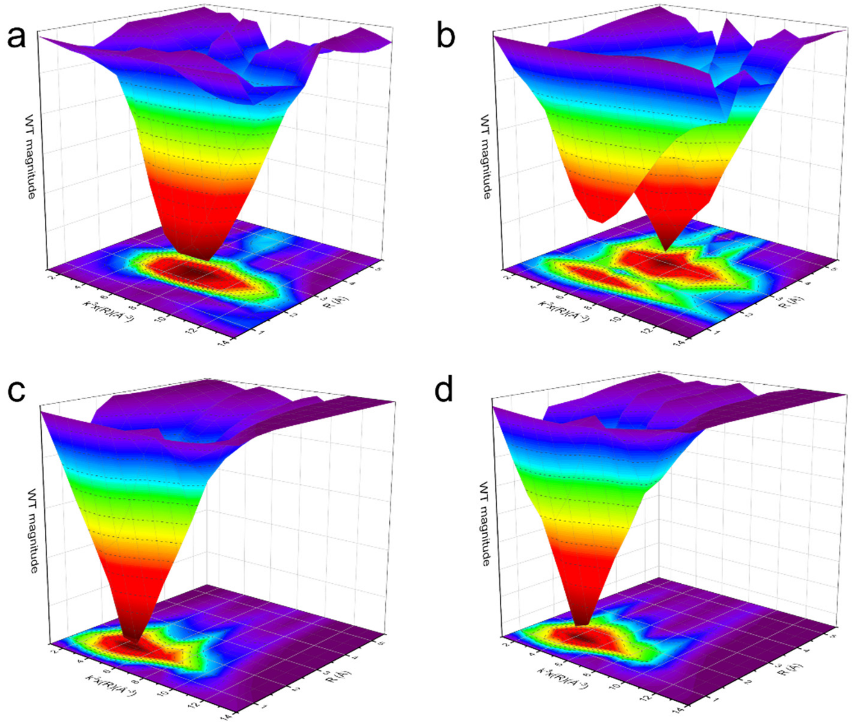

Figure S76. WT spectra of (a) Zn foil, (b) ZnO, (c) Zn-P-N-C and (d) Zn-N-C.

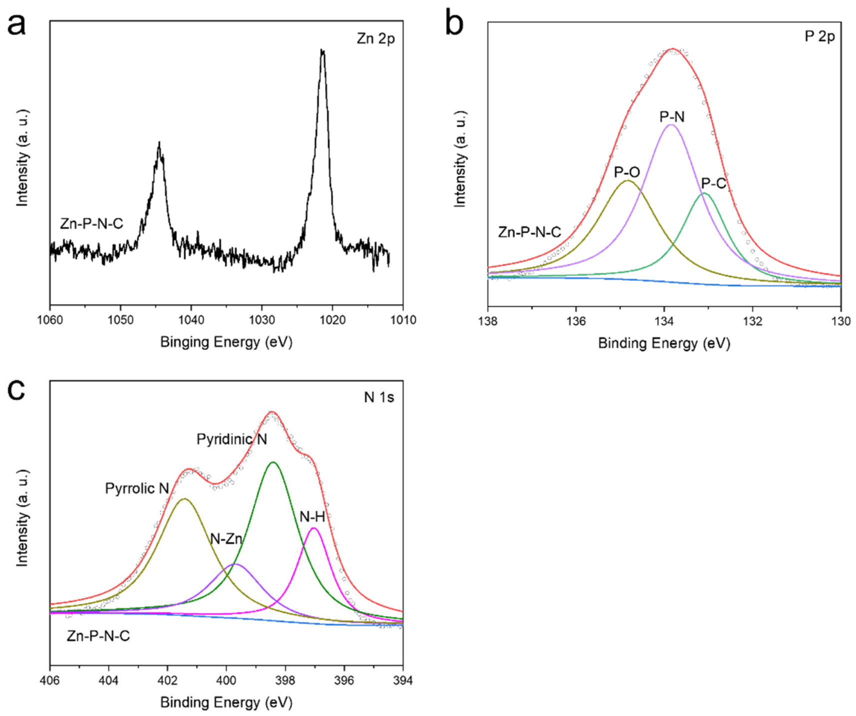

Figure S77. XPS spectra of Zn-P-N-C. (a) Zn 2p. (b) P 2p. (c) N 1s.

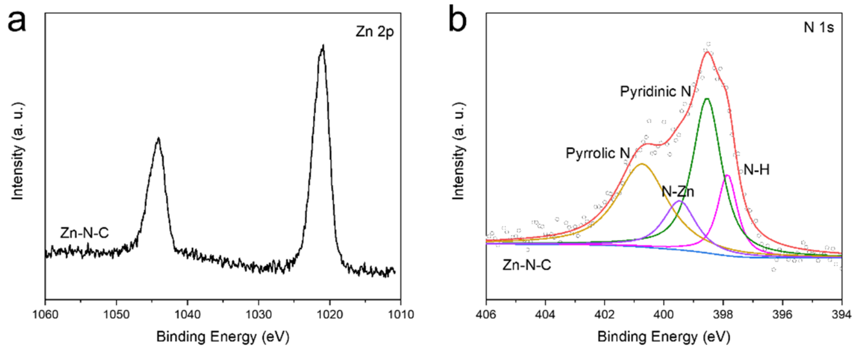

Figure S78. XPS spectra of Zn-N-C. (a) Zn 2p. (b) N 1s.

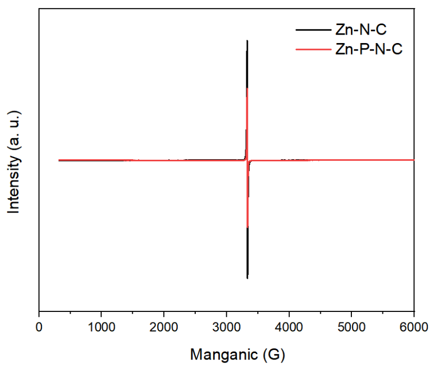

Figure S79. ESR spectra of Zn-P-N-C and Zn-N-C.

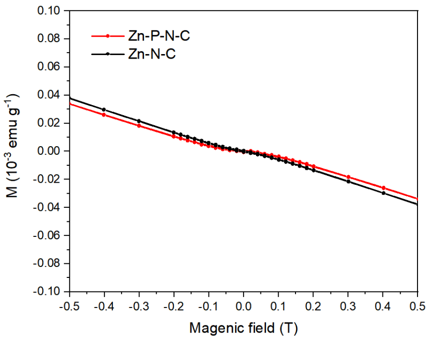

Figure S80. M-H curves of Zn-P-N-C and Zn-N-C.

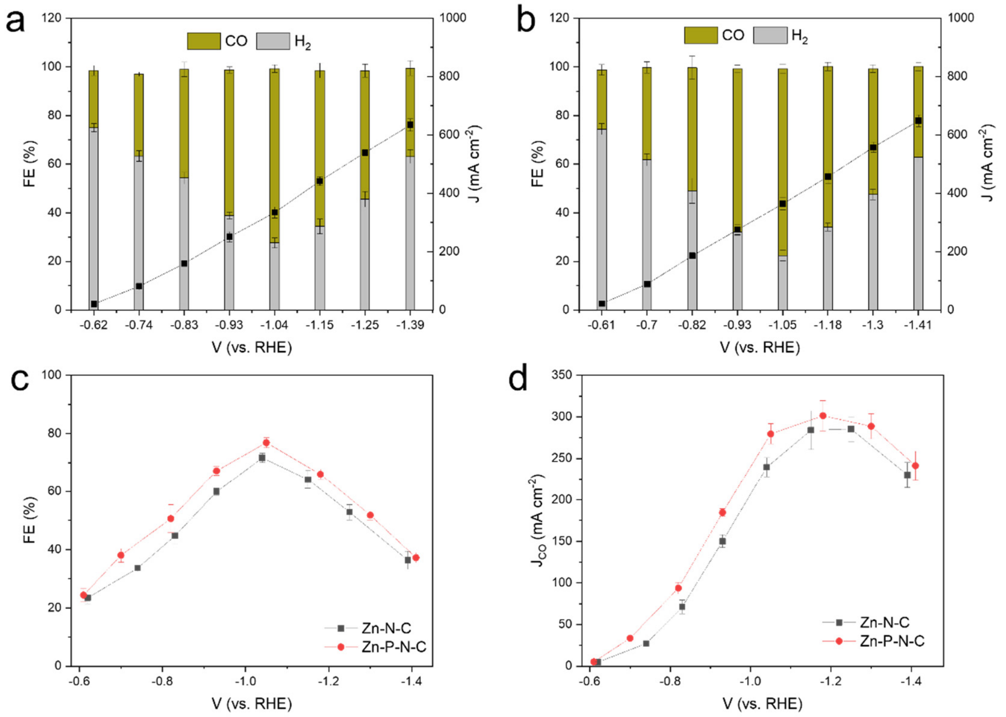

Figure S81. Potential-dependent Faradaic efficiency of each product and total current density over (a) Zn-N-C and (b) Zn-P-N-C. (c) CO FEs and (d) partial current densities of Zn-P-N-C and Zn-N-C.

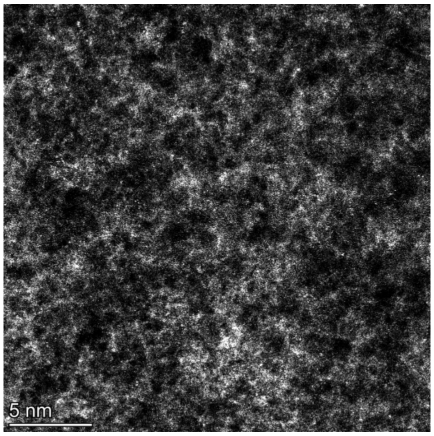

Figure S82. AC-ADF-STEM image of Zn-P-N-C after CO_2R.

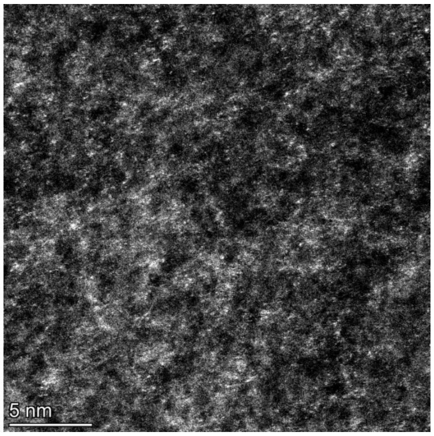

Figure S83. AC-ADF-STEM image of Zn-N-C after CO_2R.

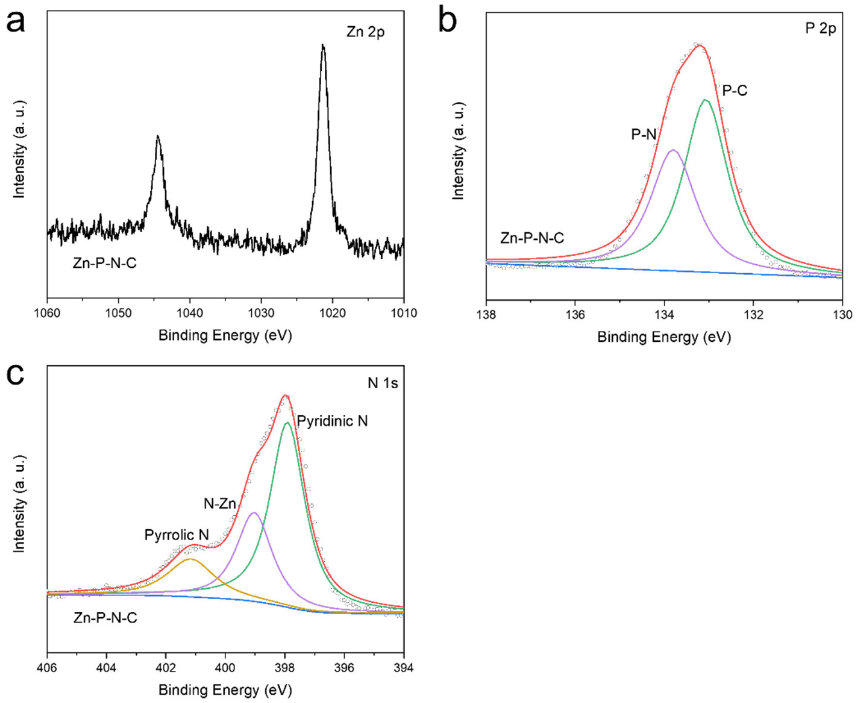

Figure S84. XPS spectra of Zn-P-N-C after CO_2R. (a) Zn 2p. (b) P 2p. (c) N 1s.

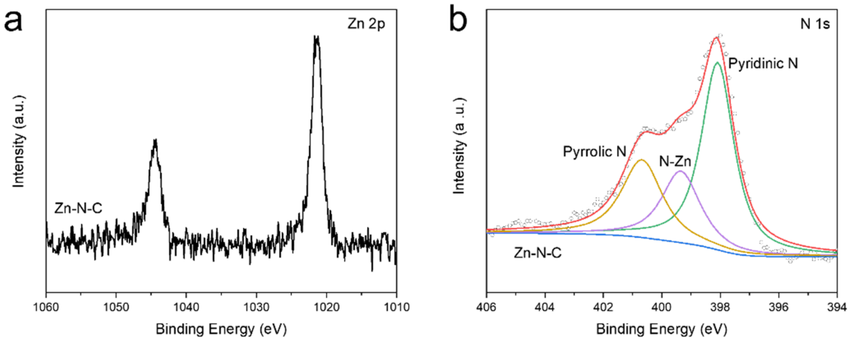

Figure S85. XPS spectra of Zn-N-C after CO_2R. (a) Zn 2p. (b) N 1s.

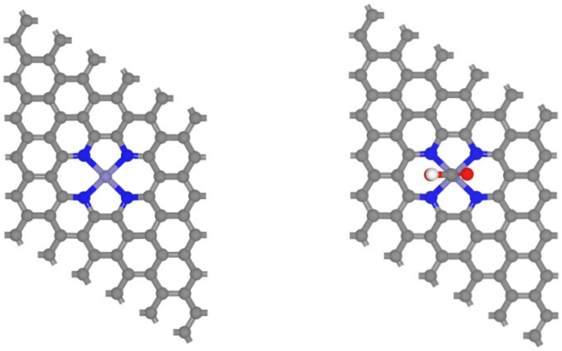

Figure S86 (labels): * ; *COOH

Figure S86. The optimized structure of Fe-N-C corresponding to the optimal adsorption model of CO_2R intermediates on the Fe-N-C surface.

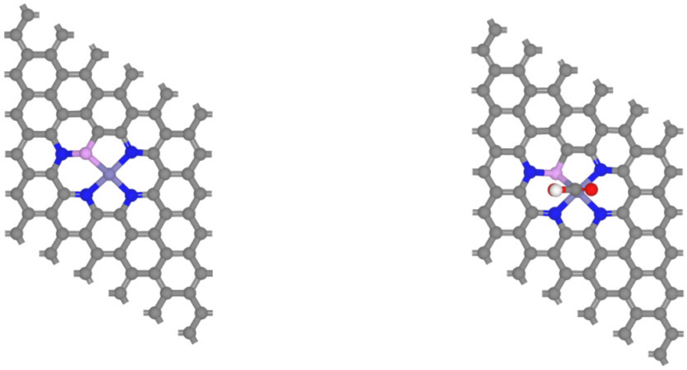

*   *COOH

Figure S87. The optimized structure of Fe-P-N-C (1) corresponding to the optimal adsorption model of CO_2R intermediates on the Fe-P-N-C (1) surface.

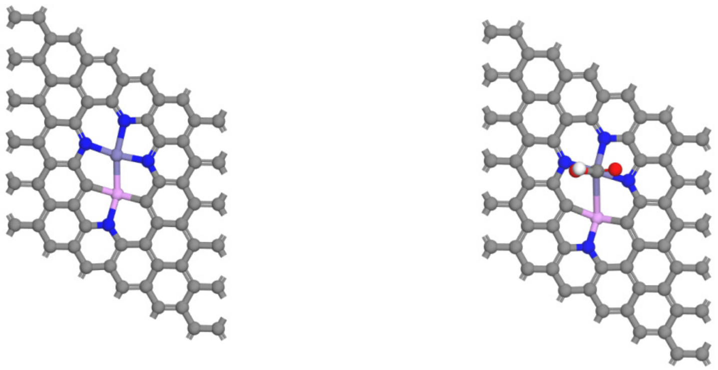

* *COOH

Figure S88. The optimized structure of Fe-P-N-C(2) corresponding to the optimal adsorption model of CO_2R intermediates on the Fe-P-N-C(2) surface.

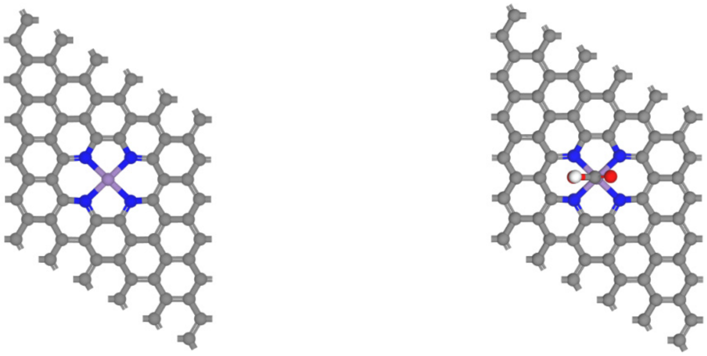

*   *COOH

Figure S89. The optimized structure of Mn-N-C corresponding to the optimal adsorption model of CO_2R intermediates on the Mn-N-C surface.

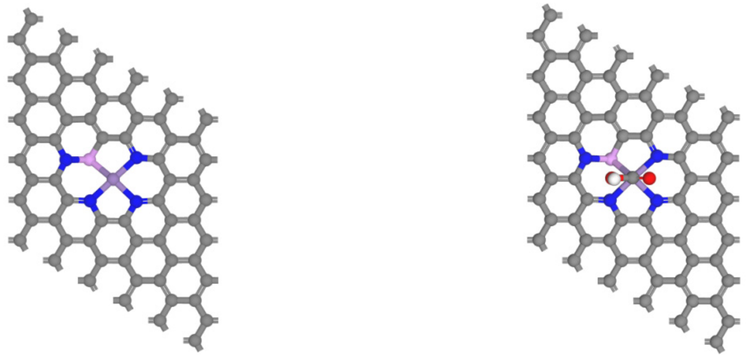

*   *COOH

Figure S90. The optimized structure of Mn-P-N-C(1) corresponding to the optimal adsorption model of CO_2R intermediates on the Mn-P-N-C(1) surface.

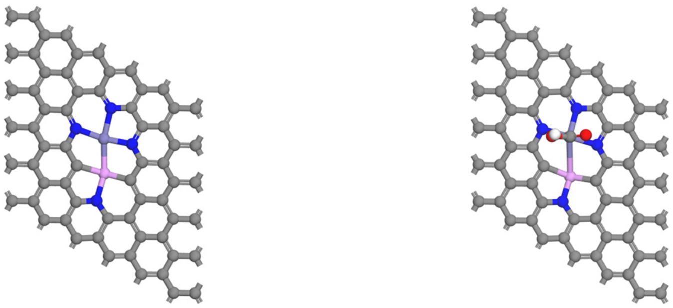

* *COOH

Figure S91. The optimized structure of Mn-P-N-C(2) corresponding to the optimal adsorption model of CO_2R intermediates on the Mn-P-N-C(2) surface.

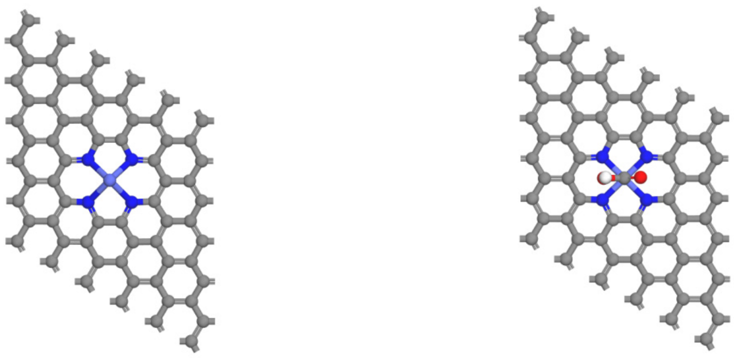

* *COOH

Figure S92. The optimized structure of Co-N-C corresponding to the optimal adsorption model of CO_2R intermediates on the Co-N-C surface.

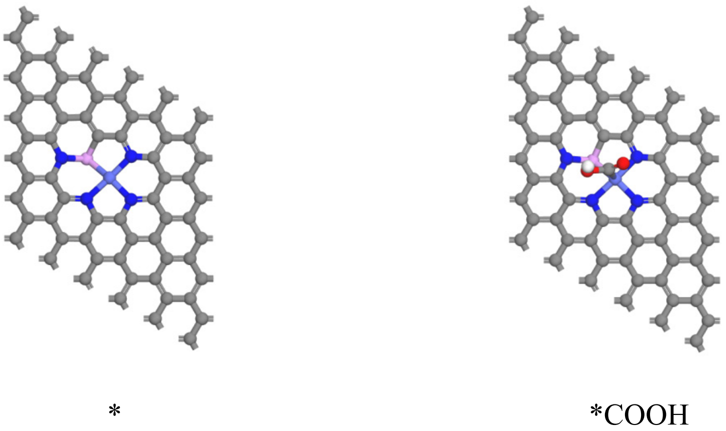

Figure S93. The optimized structure of Co-P-N-C(1) corresponding to the optimal adsorption model of CO_2R intermediates on the Co-P-N-C(1) surface.

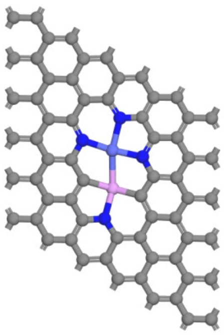

*

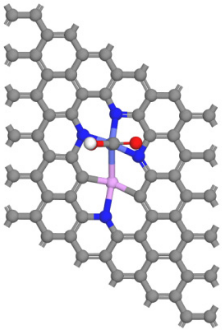

*COOH

Figure S94. The optimized structure of Co-P-N-C(2) corresponding to the optimal adsorption model of CO_2R intermediates on the Co-P-N-C(2) surface.

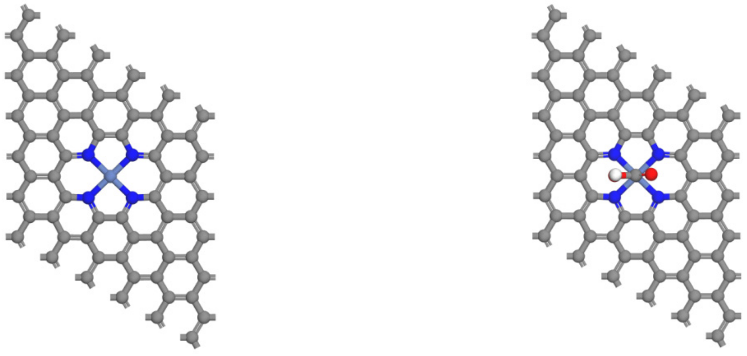

* *COOH

Figure S95. The optimized structure of Ni-N-C corresponding to the optimal adsorption model of CO_2R intermediates on the Ni-N-C surface.

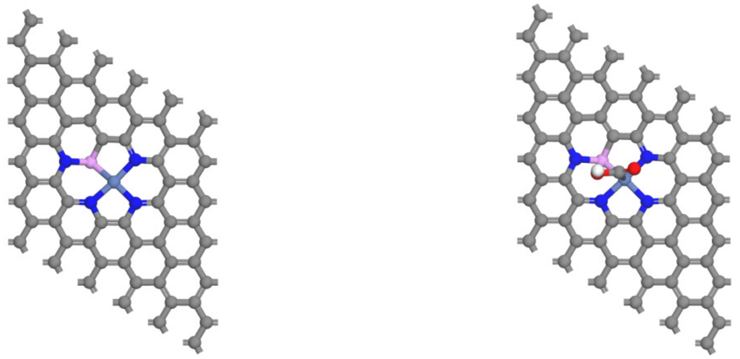

* *COOH

Figure S96. The optimized structure of Ni-P-N-C(1) corresponding to the optimal adsorption model of CO_2R intermediates on the Ni-P-N-C(1) surface.

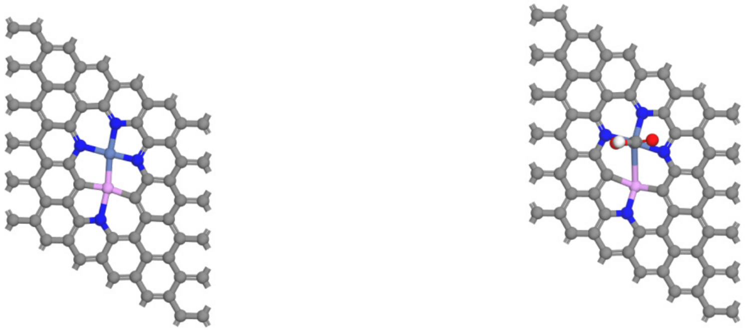

*   *COOH

Figure S97. The optimized structure of Ni-P-N-C(2) corresponding to the optimal adsorption model of CO_2R intermediates on the Ni-P-N-C(2) surface.

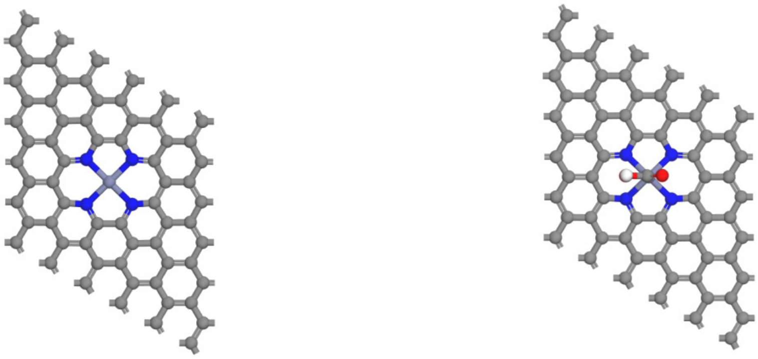

*   *COOH

Figure S98. The optimized structure of Zn-N-C corresponding to the optimal adsorption model of CO_2R intermediates on the Zn-N-C surface.

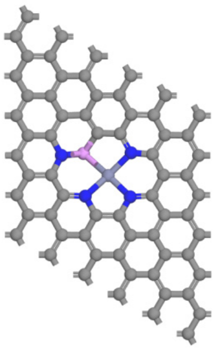

*

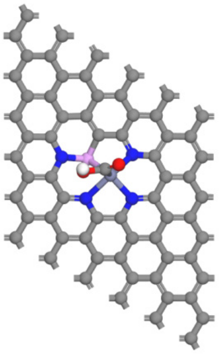

*COOH

Figure S99. The optimized structure of Zn-P-N-C(1) corresponding to the optimal adsorption model of CO_2R intermediates on the Zn-P-N-C(1) surface.

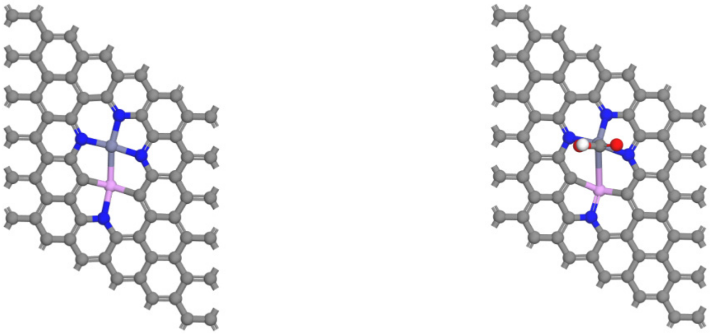

*   *COOH

Figure S100. The optimized structure of Zn-P-N-C(2) corresponding to the optimal adsorption model of CO_2R intermediates on the Zn-P-N-C(2) surface.

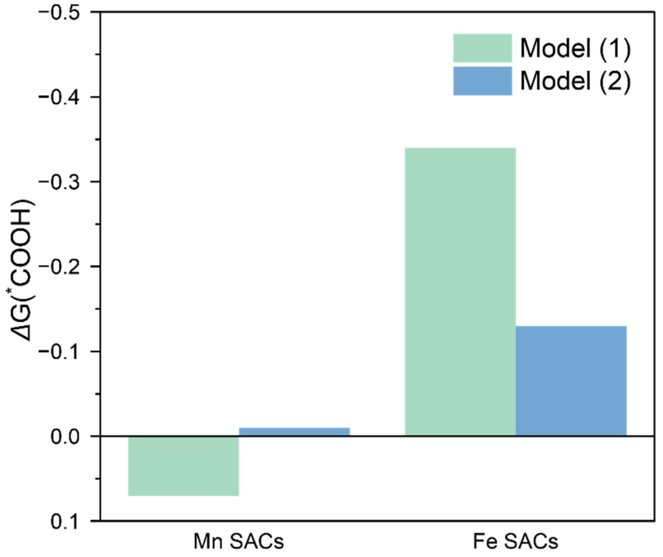

Figure S101. Comparison of the free energy change (ΔG(*COOH)) of *COOH formation in Fe and Mn SACs before and after P doping under two models, ΔG(*COOH) = G(*COOH, after P) - G(*COOH, before P).

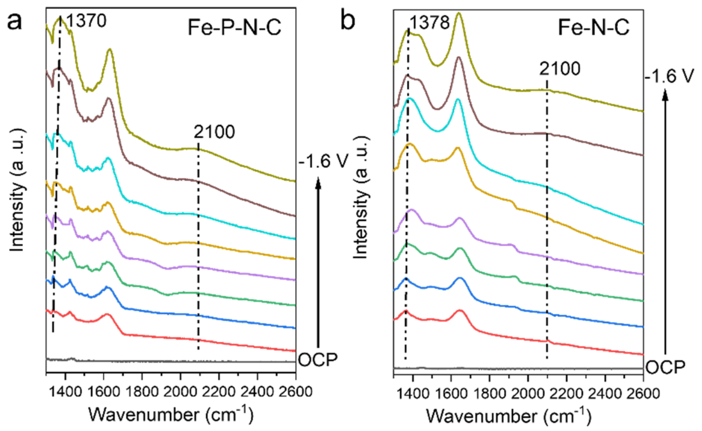

Figure S102. *In-situ* ATR-SEIRAS results of (a) Fe-P-N-C and (b) Fe-N-C. We performed these tests to investigate the mechanism. The peaks around ~1370 and ~2100 cm^{-1} could be assigned to *COOH and *CO, respectively.[1,2] The adsorbed band of *COOH locates at ~1370 cm^{-1} on Fe-P-N-C, lower than that of 1378 cm^{-1} on Fe-N-C, means the enhanced adsorption of *COOH after P incorporation. Meanwhile, the obvious signal of *CO on Fe-P-N-C than Fe-N-C means more *CO generated. In addition, the weaker signal of *COOH could be caused by higher reaction rate from *COOH to CO on Fe-P-N-C than Fe-N-C.

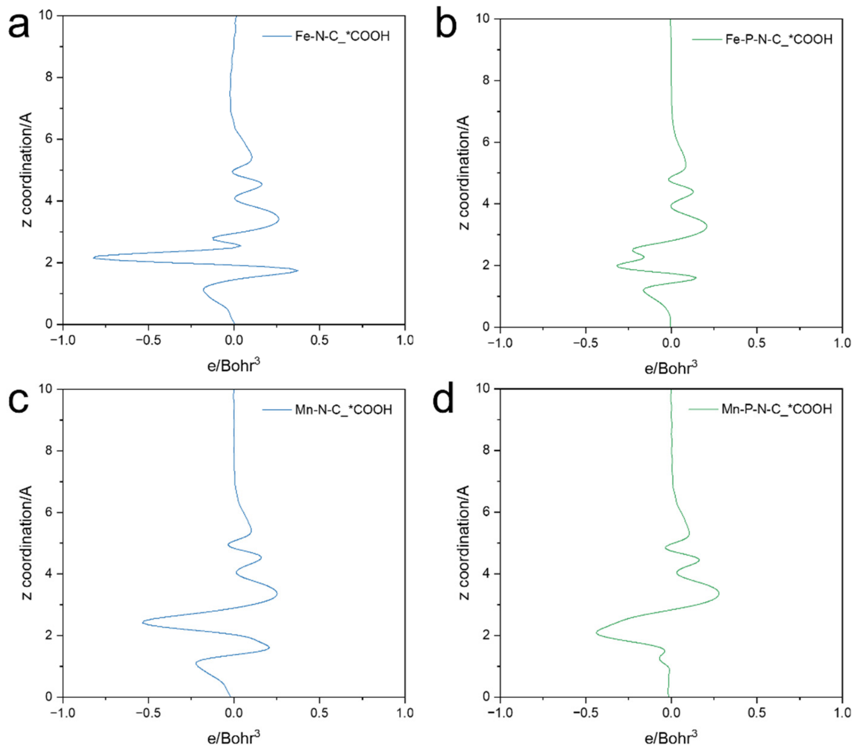

Figure S103. The average charge density difference along the *Z* axis between the *COOH of (a) Fe-N-C, (b) Fe-P-N-C, (c) Mn-N-C and (d) Mn-P-N-C.

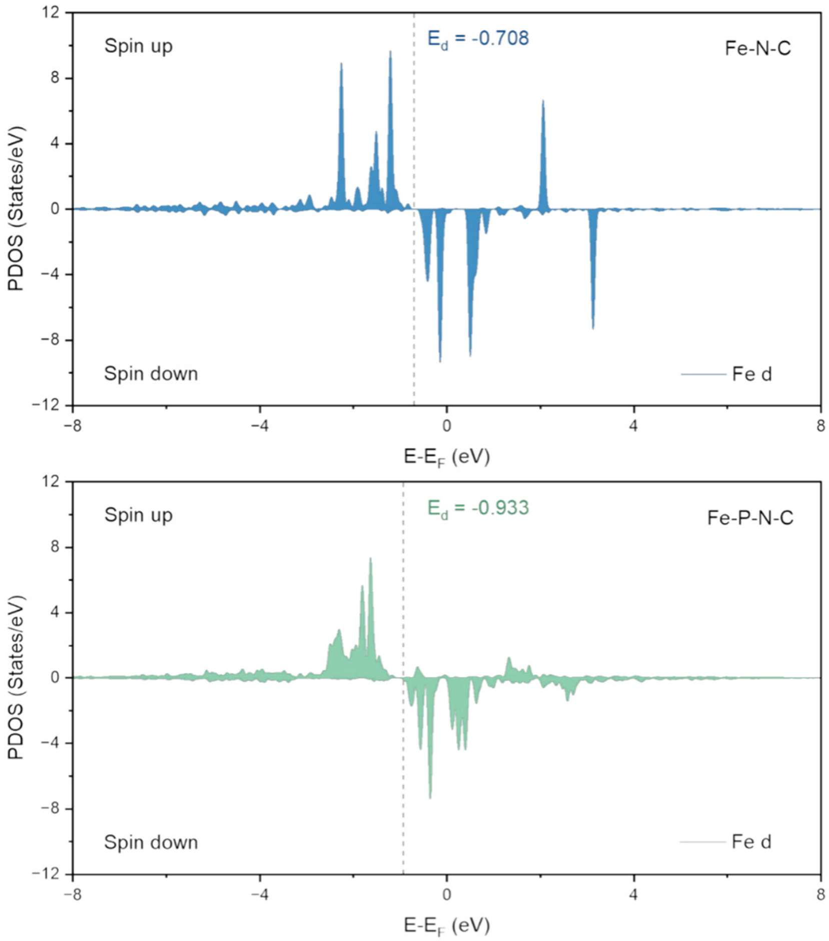

Figure S104. PDOS of Fe 3d-bands for Fe-N-C and Fe-P-N-C; corresponding d-band centers are denoted by dashed lines.

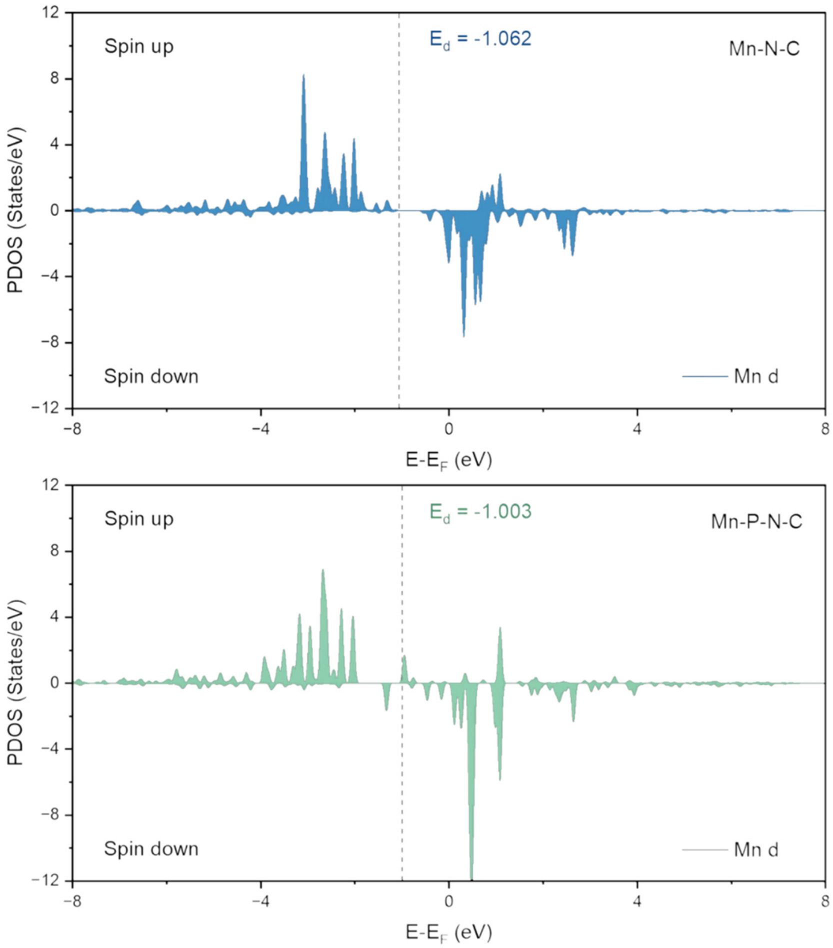

Figure S105. PDOS of Mn 3d-bands for Mn-N-C and Mn-P-N-C; corresponding d-band centers are denoted by dashed lines.

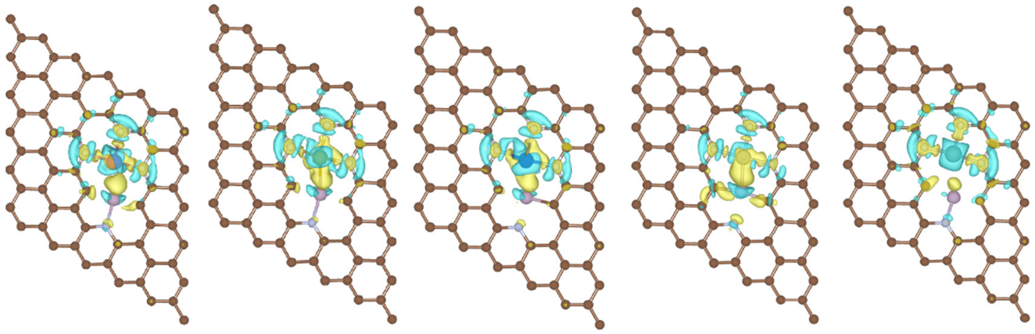

Mn-P-N-C(2) 1.21 |e|

Fe-P-N-C(2) 1.00 |e|

Co-P-N-C(2) 0.75 |e|

Ni-P-N-C(2) 0.64 |e|

Zn-P-N-C(2) 1.10 |e|

Figure S106. The charge density differences of M-P-N-C (yellow: electron accumulation; cyan: electron depletion). The isosurfaces level is set as 0.004 e/bohr^3.

Table S1. The mass loading of as-synthesized single atom samples detected by ICP-MS.

Catalyst	Loading (wt%)
Fe-P-N-C	1.67
Fe-N-C	1.80
Co-P-N-C	1.26
Co-N-C	1.60
Ni-P-N-C	1.25
Ni-N-C	1.20
Mn-P-N-C	1.26
Mn-N-C	1.38
Zn-P-N-C	0.94
Zn-N-C	1.05

Table S2. The structural parameters of as-synthesized single atom samples extracted from EXAFS fitting.

	Path	N	σ^2 (Å^2)	$\triangle E_0$ (eV)	R_f (%)
Fe-N-C	Fe-N	3.45	0.0045 (0.0004)	1.04 (0.21)	0.9
Fe-P-N-C	Fe-N	2.46	0.0049 (0.0007)	5.30 (2.50)	1.1
	Fe-P	0.68	0.0030 (0.0003)	-0.06 (0.03)	
Co-N-C	Co-N	3.75	0.0036 (0.0002)	7.58 (2.75)	1.8
Co-P-N-C	Co-N	2.37	0.0054 (0.0023)	2.06 (0.61)	1.5
	Co-P	0.54	0.0060 (0.0028)	0.49 (0.45)	
Ni-N-C	Ni-N	3.54	0.0031 (0.0006)	-1.36 (0.82)	0.8
Ni-P-N-C	Ni-N	2.70	0.0073 (0.0015)	0.67 (0.06)	1.6
	Ni-P	0.63	0.0046 (0.0007)	-9.90 (0.67)	
Mn-N-C	Mn-N	3.90	0.0032 (0.0004)	2.84 (1.80)	1.1
Mn-P-N-C	Mn-N	2.73	0.0058 (0.0013)	2.60 (0.21)	1.2
	Mn-P	0.48	0.0072 (0.0030)	4.05 (1.08)	
Zn-N-C	Zn-N	3.80	0.0028 (0.0005)	-5.10 (3.80)	1.0
Zn-P-N-C	Zn-N	2.50	0.0050 (0.0040)	-1.00 (0.46)	1.4
	Zn-P	0.61	0.0064 (0.0024)	-8.40 (2.80)	

where R is the distance between absorber and backscatter atoms, N is the coordination number, σ^2 is Debye–Waller factor, $\triangle E_0$ is the edge-energy shift, and R_f is the goodness of fit.

Table S3. Comparison of CO_2R performance among as-synthesized single atom samples and ever-reported catalysts.

Catalyst	FE_{CO} (%)	J_{CO} (mA cm^{-2})	Electrolyte	Stability test (h)	Ref.
Fe-P-N-C	96	580	1.0 M KOH	50	This work
Fe-N-C	93	415	1.0 M KOH	-	This work
Co-P-N-C	94	433	1.0 M KOH	-	This work
Co-N-C	91	368	1.0 M KOH	-	This work
Ni-P-N-C	97	567	1.0 M KOH	-	This work
Ni-N-C	93	497	1.0 M KOH	-	This work
Mn-P-N-C	79	382	1.0 M KOH	-	This work
Mn-N-C	75	362	1.0 M KOH	-	This work
Zn-P-N-C	77	301	1.0 M KOH	-	This work
Zn-N-C	72	285	1.0 M KOH	-	This work
Fe^{3+}-N-C	93	94	0.5 M $KHCO_3$	12	*Science* **2019**, *364*, 1091-1094.
CoPc/Fe-N-C	85	276	1.0 M KOH	20	*Adv. Mater.* **2019**, *31*, 1903470.
CoPc-CN/CNT	94	31	1.0 M KOH	10	*ACS Energy Lett.* **2018,** *29*, 3, 2527.
Ni-N-C-900	98	391	1.0 M KOH	70	*Energy Environ. Sci.* **2022**, *15*, 2108-2119.
NiPc-OMe MDE	99	400	1.0 M $KHCO_3$	40	*Nat. Energy* **2020**, *5*, 684.
NiSA/PCFM	83	337	0.5 M $KHCO_3$	120	*Nat. Commun.* **2020**, *11*, 593.
Ni-N-C	89	225	1.0 M $KHCO_3$	10	*Energy Environ. Sci.* **2019,** *12*, 640-647.
NiNP/NG	96	204	1.0 M $KHCO_3$	-	*Front. Catal.* **2022**, *2*, 915971.
Ni SAC-1000/CP	98	148	0.5 M $KHCO_3$	24	*J. Mater. Chem. A*, **2024**, *12*, 11090-11100.
Co3Zn1PPc	90	212	1.0 M KOH	-	*CCS Chem.* **2023**, *5*, 1130-1143.

Fe-N-C-Si	95	237	1.0 M KOH	9	*Research* **2023,** *6*, 0079.
Cu/Ni-NC	99	490	1.0 M KOH	25	*Adv. Mater.* **2023**, *35*, 2209590.
Sn-NMC-1000	92	130	1.0 M KOH	12	*CCS Chem.* **2023**, *5*, 2415-2425.
Ni-SAC-250	99	400	0.1 M K_2SO_4 $+H_2SO_4$	25	*Nat. Commun.* **2024**, *15*, 1719.
Ni-SAC-350	88.5	88.5	0.1 M K_2SO_4 $+H_2SO_4$	-	*Nat. Commun.* **2024**, *15*, 1719.
Co-SAC-250	56	56	0.1 M K_2SO_4 $+H_2SO_4$	-	*Nat. Commun.* **2024**, *15*, 1719.
Fe/NG-750	80	2.6	0.1 M KCO_3	10	*Adv. Energy Mater.* **2018**, *8*, 1703487.
Ni-N-C	82	410	0.5 M K_2SO_4 $+H_2SO_4$	8	*Energy Environ. Sci.* **2023**, *16*, 1502-1510.
Ni-SAC	91	350	1.0 M KOH	50	*Nano Res.* **2023**, *16*, 2003-2010.
Ni-NC	100	300	1.0 M KOH	8	*Adv Energy Mater.* **2022**, *12*, *2201843.*
Fe-S_1N_3	100	80	0.5 M $KHCO_3$	40	*Angew. Chem. Int. Ed.* **2023**, *63*, *e202318246.*
Ag_1-N_3 /PCNC	95	7.6	0.1 M $KHCO_3$	40	*ACS Appl. Mater. Interfaces* **2021**, 13, 17736-7744.
In_2O_3-Ag	90	102.6	0.5 M $KHCO_3$	24	*ACS Sustainable Chem. Eng.* **2024**, *12*, 9231-9238.

Table S4. Zero point of model Fe-N-C, Fe-P-N-C, Mn-N-C and Mn-P-N-C adsorbates.

Structure	*COOH
Fe-N-C	0.55
Fe-P-N-C	0.53
Mn-N-C	0.52
Mn-P-N-C	0.51

Table S5. The comparison of Fe-N-C structure energy with MAGMOM.

	G*/eV	MAGMOM
1	-653.275	-1.9989
2	-653.273	-1.9989
3	-653.161	-1.9953
4	-653.057	2.0001
5	-653.054	2.0001
6	-652.760	4.0009
7	-652.760	-4.0010
8	-652.760	-4.0009
9	-652.687	0.0000
10	-652.626	0.0118

Table S6. The comparison of Fe-P-N-C structure energy with MAGMOM.

	G*/eV	MAGMOM
1	-647.456	-2.6070
2	-647.453	2.6038
3	-647.439	1.5590
4	-647.434	-1.5577
5	-647.342	1.6161
6	-647.341	-1.6147
7	-647.339	1.6166
8	-647.326	1.6299
9	-647.303	-1.6330
10	-646.721	0.4717

Table S7. The comparison of Mn-N-C structure energy with MAGMOM.

	G*/eV	MAGMOM
1	-654.197	4.5000
2	-654.176	4.7780
3	-654.176	-4.7780
4	-654.175	-4.7780
5	-654.174	4.7778
6	-653.311	0.9997
7	-653.309	-0.9997
8	-653.153	0.9942
9	-653.148	1.0144
10	-653.037	-0.9964

Table S8. The comparison of Mn-P-N-C structure energy with MAGMOM.

	G*/eV	MAGMOM
1	-648.762	3.9931
2	-648.762	3.9930
3	-648.759	-3.9931
4	-648.758	3.9931
5	-648.757	3.9930
6	-648.754	3.9931
7	-648.753	3.9930
8	-648.753	-3.9931
9	-647.082	-0.0152
10	-647.075	0.0166

References

[1] P. Wei, H. Li, R. Li, Y. Wang, T. Liu, R. Cai, D. Gao, X. Wang, X. Bao, *Small* **2023**, *19*, 2300856.

[2] X. Qin, S. Zhu, F. Xiao, L. Zhang, M. Shao, *ACS Energy Lett*. **2019**, *4*, 1778.
